# Reported outcomes in patients with iron deficiency or iron deficiency anemia undergoing major surgery: a systematic review of outcomes

**DOI:** 10.1186/s13643-023-02431-x

**Published:** 2024-01-02

**Authors:** Stephanie Stangl, Maria Popp, Stefanie Reis, Magdalena Sitter, Lena Saal-Bauernschubert, Selina Schießer, Peter Kranke, Suma Choorapoikayil, Stephanie Weibel, Patrick Meybohm

**Affiliations:** 1 Department for Anaesthesiology, Intensive Care, Emergency and Pain Medicine, University Hospital Würzburg, Oberdürrbacher Straße 6, 97080 Würzburg, Germany; 2 Department for Anaesthesiology, Intensive Care and Pain Therapy, Goethe University, University Hospital Frankfurt, Frankfurt, Germany

**Keywords:** Iron deficiency, Iron deficiency anemia, Core outcome set, Outcome reporting, Data harmonization, Preoperative setting, Perioperative setting, Surgery

## Abstract

**Background:**

Iron deficiency (ID) is the leading cause of anemia worldwide. The prevalence of preoperative ID ranges from 23 to 33%. Preoperative anemia is associated with worse outcomes, making it important to diagnose and treat ID before elective surgery. Several studies indicated the effectiveness of intravenous iron supplementation in iron deficiency with or without anemia (ID(A)). However, it remains challenging to establish reliable evidence due to heterogeneity in utilized study outcomes. The development of a core outcome set (COS) can help to reduce this heterogeneity by proposing a minimal set of meaningful and standardized outcomes. The aim of our systematic review was to identify and assess outcomes reported in randomized controlled trials (RCTs) and observational studies investigating iron supplementation in iron-deficient patients with or without anemia.

**Methods:**

We searched MEDLINE, CENTRAL, and ClinicalTrials.gov systematically from 2000 to April 1, 2022. RCTs and observational studies investigating iron supplementation in patients with a preoperative diagnosis of ID(A), were included. Study characteristics and reported outcomes were extracted. Outcomes were categorized according to an established outcome taxonomy. Quality of outcome reporting was assessed with a pre-specified tool. Reported clinically relevant differences for sample size calculation were extracted.

**Results:**

Out of 2898 records, 346 underwent full-text screening and 13 studies (five RCTs, eight observational studies) with sufficient diagnostic inclusion criteria for iron deficiency with or without anemia (ID(A)) were eligible. It is noteworthy to mention that 49 studies were excluded due to no confirmed diagnosis of ID(A). Overall, 111 outcomes were structured into five core areas including nine domains. Most studies (92%) reported outcomes within the ‘blood and lymphatic system’ domain, followed by “adverse event” (77%) and “need for further resources” (77%). All of the latter reported on the need for blood transfusion. Reported outcomes were heterogeneous in measures and timing. Merely, two (33%) of six prospective studies were registered prospectively of which one (17%) showed no signs of selective outcome reporting.

**Conclusion:**

This systematic review comprehensively depicts the heterogeneity of reported outcomes in studies investigating iron supplementation in ID(A) patients regarding exact definitions and timing. Our analysis provides a systematic base for consenting to a minimal COS.

**Systematic review registration:**

PROSPERO CRD42020214247

**Supplementary Information:**

The online version contains supplementary material available at 10.1186/s13643-023-02431-x.

## Background

The World Health Organization (WHO) defines anemia as hemoglobin (Hb) levels <13.0 g/dL in men and <12.0 g/dL in women [1]. Iron deficiency (ID) is still the top-ranking cause of anemia in the general population worldwide [2]. In preoperative patients, the prevalence of ID ranges from 23 to 33% [3, 4], with a wide variation between surgical fields (e.g., gynecology (59%), plastic surgery (11%)) [4]. Preoperative anemia is associated with an increased risk of allogeneic blood transfusions, length of hospital stay, morbidity, and mortality [4, 5] making ID an important target to diagnose and treat before elective surgery [6]. For the detection of ID, serum ferritin (<15 μg/L) as a marker in healthy individuals and in combination with C reactive protein (CRP) in patients with inflammatory conditions (serum ferritin <70 μg/L and CRP >5 mg/L) is recommended in the WHO’s guideline from 2020 [7]. Nevertheless, cutoffs and markers for the diagnosis of ID differ widely across existing studies, guidelines, and indications for correction of ID [8]. For example, the recent guideline of the European Society of Cardiology (ESC) recommends the following laboratory parameters as indicators of ID: ferritin <30 ng/mL, transferrin saturation <20%, mean corpuscular volume <80 fL, mean corpuscular Hb <27 g/dL. In case of chronic kidney disease, chronic heart failure or infection ferritin <100 ng/mL or transferrin saturation <20% should be utilized [9].

First-line treatment for iron deficiency anemia (IDA) is supplementing iron intravenously or orally which is part of Patient Blood Management (PBM) programs [10, 11]. Iron supplementation can increase Hb levels in patients with IDA if started in time before an upcoming surgery. Various studies suggested that intravenous (IV) iron supplementation reduces the need for red blood cell transfusions and postoperative complications [4, 5, 12–14]. Although there is an immense amount of studies investigating preoperative anemia management, it remains challenging to establish reliable evidence on the efficacy of preoperative iron supplementation [14]. Limitations arise from the heterogeneity of iron treatment regimes, thresholds for indication of blood transfusion, and definition of anemia and ID [14].

Another limitation in many clinical scenarios stems from the heterogeneity of outcomes reported in clinical trials which hampers a direct comparison between trials [15]. To guide future trials addressing the efficacy of treatment of IDA in a preoperative setting, a core outcome set (COS) is needed. A COS represents a minimum of outcomes that should be assessed to facilitate a comparison of treatment effects between several studies. Thus, a combination of and comparison between different studies is possible and allows meaningful conclusions to be drawn [15].

Therefore, this systematic review aimed to identify and assess the scope and consistency of outcomes including definitions and measurements reported by randomized controlled trials (RCTs) and observational studies for the treatment of diagnosed pre- and perioperative ID with or without anemia in a non-perinatal setting as a first step of a COS development process for future clinical trials.

## Methods

This systematic review is reported in accordance with the Preferred Reporting Items for Systematic Reviews and Meta-Analyses (PRISMA) checklist [16]. The protocol was registered with the PROSPERO database (CRD42020214247, available from https://www.crd.york.ac.uk/prospero/display_record.php?ID=CRD42020214247) and with the Core Outcome Measures in Effectiveness Trials (COMET) registry (https://www.comet-initiative.org/Studies/Details/1704).

### Search strategy

We searched the electronic databases MEDLINE (via PubMed), Cochrane Central Register of Controlled Trials (CENTRAL) (via Cochrane Library), and ClinicalTrials.gov for published articles or registered studies from 2000 to April 1, 2022. The search in ClinicalTrials.gov was restricted to completed trials. Language was restricted to English, Spanish, and German. Our search strategy included medical subject headings (MeSH) and free text. Our search terms are provided as online Additional file 1.

### Eligibility criteria

We included RCTs and observational studies comparing iron alone or in combination with erythropoietin to standard of care (SoC), placebo, or any active comparator (e.g., iron administered by a different route) in patients aged 18 years or older, any sex, presenting with diagnosed ID, with or without anemia, and scheduled for surgery of any kind.

The original protocol defined the eligible study population as “adult participants (majority ≥18 years) with suspected or diagnosed iron deficiency with or without anemia (ID(A)) undergoing surgery” [17]. During the screening process, it became apparent that “suspected” ID as an inclusion criterion was not sufficient to identify studies aiming to correct preoperative and perioperative ID. Therefore, we amended the protocol and classified studies as eligible when the diagnosis of ID(A) was preoperatively and laboratory-confirmed in the investigated surgical patient population (studies, that investigated patients with IDA without a laboratory confirmation were excluded for the reason of “insufficient in-/exclusion criteria”). Studies comprising children or patients with anemia from other causes were excluded. Studies that investigated interventions to prevent anemia as a consequence of surgical procedures in primarily non-anemic patients, to treat anemia of other causes, or to treat anemia in non-surgical patients were excluded (as “wrong population”). Eligible interventions were pre- or intraoperative administration of oral or IV iron, iron with erythropoietin, or erythropoietin alone by any administration route. Additional SoC treatment in the intervention group was allowed as long as it was applied to the control arm as well. Only studies with at least one control group were included. Control interventions could include a placebo, SoC, no treatment, or any active comparator.

### Study selection and data extraction

Records identified via the database searches were imported to Endnote, and duplicates were removed. References were then exported to the web-based software platform Covidence (www.covidence.org) and screened by two independent reviewers (SSc, MP, SR, SW) on the title/abstract level for eligibility. For the remaining records, the full texts were retrieved and screened for eligibility. The reason for the exclusion of each study was noted. Disagreement between raters was resolved by discussion or a third person.

Studies, that were excluded with the reason of “insufficient in-/exclusion criteria” (i.e., without a laboratory confirmation of IDA), were further investigated regarding their intention and rationale. The in- and exclusion criteria from the respective RCTs are listed as online Additional file 2.

Ongoing studies were not eligible for data extraction to ensure a comprehensive description of outcomes (e.g., definition, statistical measure, questionnaire).

Study characteristics (e.g., publication details, population characteristics, intervention, and comparator description; see online Additional file 3) and reported outcomes (outcome definition and measures, instrument used to assess outcome, and time points of measurement) were extracted from included studies by one reviewer to a MS® Excel sheet and double checked by a second reviewer. Outcomes were extracted as primary and secondary as stated in the respective publication. If there was no classification provided, we considered the outcome for which the study’s sample size had been calculated or if not applicable, the first one described in the study, as the primary outcome. In addition, for studies that were registered on publicly available study registry platforms, registered outcomes were compared to outcomes reported in the respective publication about selective outcome reporting bias. Reported results were classified as “benefit” (i.e., intervention improves outcome compared to control) or “no benefit”, with or without being statistically significant. Furthermore, information on missing *p* values (“not reported”), as well as non-reported outcomes (“not applicable”), was noted. In case a sample size calculation was conducted, outcomes and clinically relevant differences stated were extracted. Finally, extracted outcomes were classified and summarized according to the outcome taxonomy by Dodd et al. [18]. This outcome taxonomy provides five core areas (i.e., death, physiological/clinical, life impact, resource use, adverse events), which can be subdivided into several outcome domains (e.g., “social functioning” as outcome domain of the core area “life impact”). A short user guide is provided here [19]. Outcomes were classified by two review authors. Any disagreement between review authors was solved by discussion. Frequencies of outcome domains and variations among included studies were assessed.

### Quality assessment

For the quality assessment on outcome definition and reporting, questions as proposed by the MOMENT study protocol [20] were used and adapted as described in Table 1.

**Table 1:** Quality assessment of outcome reporting and trial registration

Domain	Assessment criteria^a^
Methodological outcome reporting (based on MOMENT criteria [20])	1) Is the primary outcome clearly stated (in the method section)?
	2) Is the primary outcome clearly defined (method section/protocol/register) so that another researcher would be able to reproduce its measurement?
	3) Are the secondary outcomes clearly stated (in the method section)?
	4) Are the secondary outcomes clearly defined (method section/protocol/register)?
	5) Do the authors provide a rational for the use of the outcomes they have selected?
	6.1) Are all defined primary outcomes and secondary outcomes reported which are defined in the method section?
	6.2) Are reported outcomes limited to the outcomes defined (in the method section)?
Trial registration	7.1) Was the trial prospectively registered?
	7.2) Are all (registered) primary and secondary outcomes reported?
	7.3) Are reported outcomes limited to those registered?
	7.4) Is there no sign of selective outcome reporting (e.g., change in primary or secondary outcome, new primary outcome, omission of primary outcome)?

^a^Allowed categories for quality assessment were yes/no/not applicable

Two independent reviewers (StSt, MP, LSB, SR, SW) rated the studies based on each question with the allowed categories “yes/no/not applicable”. Disagreement was resolved by discussion or a third person.

We decided not to present an overall score, since there is no evidence on weighting the questions and cutoff values. For transparency, we assessed each question per study.

## Results

**Fig. 1:**
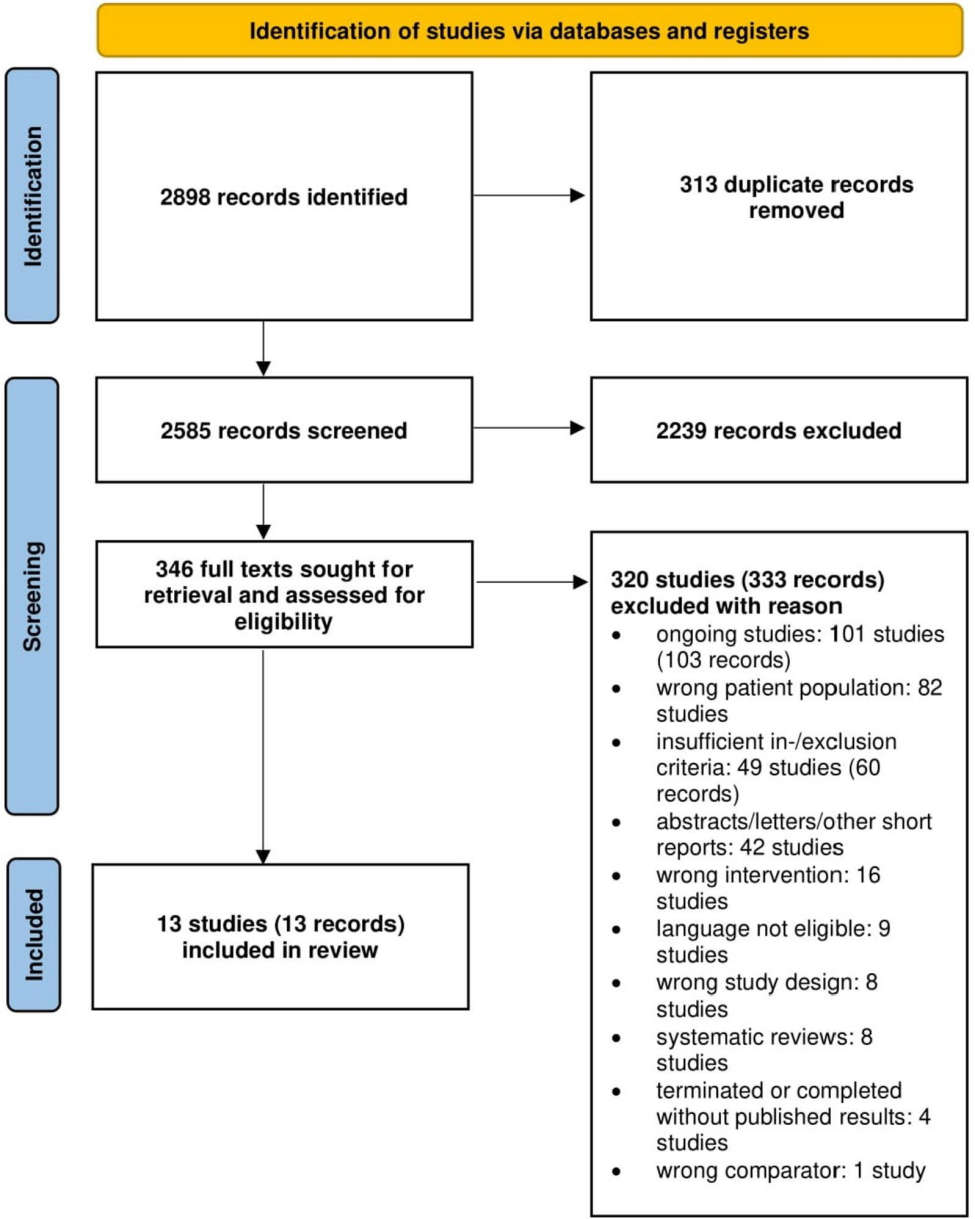
PRISMA flow chart [16]

Our search strategy retrieved 2898 records. After removing 313 duplicates, titles and abstracts from 2585 records were screened. Eligibility of 346 full texts was assessed, and 13 studies, only comprising patients with diagnostically confirmed ID(A), were included in the review (see Fig. 1).

Three hundred twenty studies representing 333 records were excluded with reason, of which 101 studies were protocols or registry entries of ongoing studies. Further, 82 studies were excluded, because iron with or without erythropoietin was supplemented in non-surgical anemic patients, or as prevention of anemia expected to develop after surgery. Further, 49 studies were excluded due to a lack of diagnostically confirmed IDA due to “insufficient in-/exclusion criteria.” Of those twenty RCTs stated having investigated preoperatively anemic patients (online Additional file 2). Other reasons for exclusion were wrong publication types such as abstracts and letters (42 studies), wrong intervention (16 studies), non-eligible languages (nine studies), wrong study design (eight studies), systematic reviews (eight studies), terminated or completed studies without results (four studies), and one study with wrong comparator.

In total, 13 studies met our inclusion criteria. The eligible 13 studies comprised five RCTs [21–25] and eight observational studies: two prospective [26, 27], five retrospective cohort studies [28–32], and one observational study [33] comparing a prospective intervention group to a historic cohort. Table 2 gives an overview of the study characteristics. If applicable, extracted data are restricted to patients with ID(A).

**Table 2:** Study characteristics of eligible studies on iron deficiency, with or without anemia

Study [registration number]	Country	Study type	Definition of anemia (Hb level)	Diagnostic criteria (iron deficiency)	Intervention group	Control group^a^	Type of surgery	Recommendation on start of iron supplementation
Abdullah-2021 [28]	Singa-pore	Retrospective cohort study	<13 g/dL	Ferritin<100 μg/L, TSAT<20%	**IV iron (FCM)**, *n*=89	**Oral iron**, *n***=**267	Elective surgeries under general/regional anesthesia	1 month prior to surgery according to institutional protocol
D'Amato-2020 [29]	Italy	Retrospective cohort study	NA (only non-anemic ID patients included)	Ferritin<100 μg/L and normal CRP values	**IV iron (FCM)**, *n*=83	**SoC**, *n***=**62	Total hip or knee arthroplasty	4 weeks prior to surgery
Froessler-2016 [21] [ACTRN12611000387921]	Australia	RCT –Phase III	<13(m)/12(f) g/dL	Ferritin<300 μg/L, TSAT<25%	**IV iron (FCM)**, *n*=40	**SoC or oral iron**, *n*=32	Major abdominal surgery	21-4 days prior to surgery (+ within 2 days after surgery)
Ionescu-2020 [30]	Romania	Retrospective cohort study	<13(m)/12(f) g/dL	Ferritin <300 μg/l, TSAT<25%	**IV iron (FCM)**, *n*=329	**ABT**, *n*=342; **SoC**, *n*=2282	not specified	Not reported
Kim-2009 [22]	South Korea	RCT –Phase IV	<9.0 g/dL	established IDA’, no further definition	**IV iron sucrose**, *n*=39	**Oral iron protein succinylate**, *n*=37	Gynecological surgery	3 weeks prior to surgery
Klein-2020 [27] [NCT02637102], [ISRCTN55032357]	UK	Prospective cohort study (CAVIAR Study)	<13(m)/12(f) g/dL	Ferritin<100 μg/L and TSAT <20%	**IV iron (FCM** or **IIM)**, *n*=68	**SoC**, *n*=75	Cardiac surgery	10 days prior to surgery
Laso-Morales-2017 [31]	Spain	Retrospective cohort study	<13 g/dL	Ferritin<300 μg/L; or TSAT<20% (if CRP>5 mg/L, and normal-to-elevated ferritin)	**IV iron sucrose** (or **FCM**)	**SoC**, *n*=76 or **oral iron**, *n*=23	Colorectal cancer resection	6-4 weeks prior to surgery
Lee-2019 [23]	South Korea	RCT – Phase NA	<10 g/dL	Ferritin<300 μl/L	**IV iron (FCM)**, *n*=52	**IV iron** **sucrose**, *n*=49	Gynecological surgery for benign diseases causing menorrhagia	Prior to surgery, not further specified
Na-2011 [24]	South Korea	RCT – Phase NA	NA – (only ID patients with Hb>10 g/dL included)	Ferritin<100 μg/L or 100-300 μg/L with TSAT<20%	**IV iron sucrose** with **EPO**, *n*=56	**SoC**, *n*=57	Bilateral total knee replacement	Prior to surgery, not further specified
Nandhra-2020 [26] [NCT02637102], [ISRCTN55032357]	UK	Prospective cohort study (CAVIAR study)	<13(m)/12(f) g/dL	Ferritin<100 μg/L and/or TSAT<20%	**IV iron,** not further specified, *n*=15	**SoC**, *n*=57	Vascular surgery	10 days prior to surgery
Scardino-2019 [32]	Italy	Retrospective cohort study	NA – (only non-anemic ID patients included)	Ferritin<100 μg/L or TSAT<20% (if CRP>3 mg/L, and normal-to-elevated ferritin)	**Oral sucrosomial iron**, *n*=100	**SoC**, *n*=100	Hip surgery	3-4 weeks prior to surgery
Shin-2020 [33]	South Korea	Prospective cohort study with historic control	<13(m)/12(f) g/dL	Ferritin<30 μg/L or 30-100 μg/L with TSAT<20%; (control group: not tested)	**IV iron (FCM)**, *n*=46	**SoC**, *n*=46	Total knee arthroplasty	4 weeks prior to surgery
Thin-2021 [25] [NCT03295851]	Singa-pore	RCT – phase IV (pilot study, PIRCAS trial)	<13(m)/12(f) g/dL	Ferritin<100 μg/L or 100–300 μg/L with TSAT<20%;	**IV iron (FCM)**, *n*=15	**Oral iron**, *n*=46	Major abdominal surgery	4 weeks (IV iron)/ 1-4 weeks (oral) prior to surgery

*ABT* allogenic blood transfusion, *CRP* C-reactive protein, *EPO* erythropoetin-b, *f* female, *FCM* ferric carboxymaltose, *Hb* hemoglobin, *IIM* iron isomaltoside, *IV iron* intravenous iron, *m* male, *n* number of participants, *NA* not applicable, *RCT* randomized controlled trial, *SoC* standard of care, *TSAT* transferrin serum iron saturation, ^a^Extracted information on control group restricted to patients with ID or IDA (except for Shin et al.)

Seven studies originated in the Asia-Pacific region (*n*=4 South Korea, *n*=2 Singapore, *n*=1 Australia) and six in Europe (*n*=2 Italy, *n*=2 UK, *n*=1 Romania, *n*=1 Spain). Anemia was defined in accordance with the WHO definition in six studies and two studies stated Hb <13 g/dL irrespective of gender. Two studies included solely ID patients without anemia [29, 32]. Kim et al. and Lee et al. set an Hb level of <9 and 10 g/dL, respectively, as definition criteria and Na et al. included only patients with Hb >10 g/dL [22–24]. Three studies (i.e., four publications) were registered within a study registry [21, 25–27]. Most studies (*n*=12 (92%)) supplemented IV iron in the intervention group. The majority of studies administered iron at least 3 weeks prior to surgery. Two publications [26, 27] (both from the CAVIAR study) supplemented iron 10 days prior to surgery. Three studies did not give a timeframe for recommended iron supplementation [23, 24, 30]. Na et al. combined IV iron with recombinant human erythropoietin-ß [24]. Solely Kim et al. used oral iron (30 mg capsule per day 3 to 4 weeks prior to surgery) as an intervention [22]. Active comparators were used in five studies (*n*=1 IV iron, *n*=1 allogenic blood transfusion, *n*=3 oral iron) [22, 23, 25, 28, 31]. SoC was utilized in eight studies and mostly consisted of no preoperative iron treatment. Oral iron as part of SoC was administered in two studies [21, 31]. Orthopedic (*n*=4) followed by gynecological and major abdominal (both *n*=2) surgeries were the most frequent surgical interventions investigated in the studies. The mean age of the intervention or control group was 42 years or older. Both studies, with mean age <50 years consisted of patients undergoing gynecological surgery [22, 23]. A table containing verbatim details from the study’s publication can be found as online Additional file 4.

### Outcome reporting

Across all 13 studies, 111 individual outcomes were reported. We summarized them into nine overall outcome domains across five core areas according to the Outcome Taxonomy by Dodd et al. [18] (see Tables 3 and 4).

**Table 3:** Core area “death”, “physiological/clinical outcomes,” and “adverse events” (taxonomy based on Dodd et al. [18])

Core area	Outcome domain	Further definition of outcome (if applicable)	No. of studies, *n* (%)	No. of studies reporting as primary outcome	Variation in reported outcome
**Death**	Mortality/survival	Mortality	5 (38) [21, 25–27, 30]	0/5	- Reported as number or percentage of patients deceased - Time points: from up to 30 days to 6 months after intervention
**Physiological/clinical outcomes**	Blood and lymphatic system outcomes	Hemoglobin (Hb) levels	12 (92) [21–27, 29–33]	6/12	- Continuous variable: reported as mean ± SD concentrations or differences in concentrations - Categorical variable: success versus failure in reaching target Hb (10g/dL); number/percentage of patients reaching Hb >10g/dL - Time points: at baseline, 28-35 days prior to surgery, at day of surgery, 1 day to 6 months postoperatively, at discharge - 2 studies reported number of patients reaching a Hb level of 10 g/dL before surgery and time needed for it
		Change in iron metabolism parameters	6 (46) [21, 22, 24, 25, 30]	1/6	- Reported as mean ± SD concentrations or differences in concentrations of iron parameters: ferritin, transferrin, transferrin saturation, iron, iron-binding capacity - Time points: up to 4 preoperatively and up to 4 postoperatively or not specified
		Blood loss	2 (15) [24, 33]	0/2	- Reported as estimated mean blood loss ± SD and blood loss in drainage - Time points: 5 and 7 days postoperatively
		Other outcomes regarding hematopoiesis	2 (15) [22, 29]	0/2	- Reported as mean ± SD or differences in number of white and red blood cells, and mean cell volume - Time points: preoperatively up to 4 weeks and up to 4 weeks postoperatively
**Adverse events**	Adverse events (AE)	See variation in reported outcome	10 (77) [21–23, 25–27, 29–31, 33]	0/10	- Reported as incidence/number of AEs with various definitions: ‘Clavien-Dindo-Classification’, ‘Common Terminology Criteria for Adverse Events’, overall complications, mild AEs, AEs with need for further intervention, serious AEs, AEs related to study drug, specific AEs (blood and lymphatic system, gastrointestinal system, immune system, infection and infestation, nervous system, respirator system, renal and urinary system, vascular system; general symptoms such as pain, nausea), or not further specified - Reported as odds ratio with 95% CI (association of factors with outcome of interest), median with IQR - Categorical analysis of subtypes of adverse events (e.g., surgical wound as subtype of infection etc.) or subdivision of Clavien Dindo Score (1/2 vs. 3a-5 vs. 3 vs. 4 vs. 5) Time points: 4 weeks preoperatively and 4 days postoperatively, preoperatively, during treatment, and up to 30 days postoperatively

*AE* adverse event, *Hb* hemoglobin, *No*. number, *SD* standard deviation.

**Table 4:** Core area “life impact” and “resource use” (taxonomy based on Dodd et al. [18])

Core area	Outcome domain	Further definition of outcome (if applicable)	No. of studies, *n* (%)	No. of studies reporting as the primary outcome	Variation in reported outcome
**Life impact**	Delivery of care	Outcomes related to the feasibility of study/intervention	3 (23) [25–27]	3/3	- Feasibility of the study reported as the number of participants recruited/enrolled, consenting, receiving study drug, and completing the study. - Time points: within 4 to 12 months, or not specified
	Global quality of life	Quality of life	6 (46) [21, 23, 25–27, 30]	0/6	- Reported as median with IQR or mean ± SD scores on various quality of life questionnaires, such as Short-Form (SF-12, SF-36), Single Question Outcome Measure (SQOM), European Quality of Life (EQ-5D-3L, EQ-5D-5L) - Time points: from baseline to 2 weeks, 30 days, 3 months, 6 months after intervention or surgery or not specified
	Physical functioning	*See variation in reported outcome*	2 (15) [26, 27]	0/2	- Reported as median with IQR or mean ± SD days and scores on various physical functioning scales, such as Days-alive-and-out-of-hospital (DAH/DAOH; 6-minute walk test (6MWT); cardiopulmonary exercise testing (CPET) Multidimensional Fatigue Inventory-20 (MFI-20) - Time points: from baseline to 2 weeks, 30 days, 3 months, 6 months after intervention or surgery or not specified
**Resource use**	Need for further intervention	Need for blood transfusion	10 (77) [21, 24–29, 31–33]	3/10	- Reported as number or percentage of patients receiving blood transfusion; number of blood units transfused or amount of transfused blood in total or per patient in mean ± SD or median with IQR; reduction in risk or odds ratio for (with 95% CI) blood transfusion; categories of units transfused used: 1–2 and 3–4 - Time points: preoperatively without further specification or at up to 30 days before surgery; during surgery; postoperatively at 5 days, 7 days, 30 days until discharge or not further specified; other time points: during hospital stay; from overall from recruitment to discharge or not specified
		Iron treatment	1 (8) [21]	0/1	- Reported as number of patients requiring continued iron treatment at the time of discharge from hospital
	Hospital	Use of hospital resources	8 (62) [21, 25–29, 31, 32]	0/8	- Reported as mean ± SD or median with IQR or with minimum and maximum of length of hospital stay and length of ICU stay; number of patients who need readmission to hospital or ICU admission - Time points: until discharge, within 30 days or not specified
	Economic	Cost savings per patient	1 (8) [32]	0/1	- Reported as the total difference of costs per patient between groups calculated from costs for transfusion, iron supplementation, and hospitalization - Time points: from admission to end of hospital stay

*ICU* intensive care unit, *IQR* interquartile range, *SD* standard deviation

### Mortality

Mortality (core area “death”) was reported as the number or percentage of patients deceased ranging from up to 30 days to 6 months after intervention in five studies (38%) [21, 25–27, 30]. No study reported mortality as the primary outcome. This outcome naturally resulted in homogeneous reporting regarding its definition; however, measured time points showed high variance between studies (see Table 3).

### Clinical outcomes

The most frequently reported outcome domains were “blood and lymphatic system outcomes” in the core area of “clinical outcomes”. Twelve [21–27, 29–33] of 13 studies (92%) investigated the influence of treatment on Hb levels, of which six studies defined this as their primary study outcome. The measurement of the outcome was consistently reported as mean ± standard deviation (SD) of Hb concentrations or mean differences between concentrations. However, the timeframe after baseline measurement in which Hb changes were investigated varied widely from 4 weeks before surgery up to 6 months after surgery. Some studies also reported this outcome as the success rate of patients reaching a certain Hb level before surgery and the time needed for the respective success.

Six (46%) studies [21, 22, 24, 25, 30] reported a change in iron metabolism parameters as mean ± SD concentrations or differences in concentrations of one, several, or all of the following: serum iron concentrations, serum ferritin concentrations, transferrin saturation, or iron-binding capacity. Time points of measurement ranged from 4 weeks before and 4 weeks after surgery, or there was no time point specified in the study.

Other outcomes, from the core area “clinical outcomes”, such as renal or infection outcomes were reported in fewer studies and none of them as a primary outcome (see Table 3).

### Adverse events

Ten (77%) studies [21–23, 25–27, 29–31, 33] reported adverse events, although never as primary outcome. The variance was seen across all studies regarding definitions and timing of measurements as described in Table 3. Regarding the prior definition of outcome assessment, we only found two studies providing a reference to an official scale or definition of how adverse events should be recorded [23, 25]. Six studies stated having recorded adverse events or side effects “related to the study intervention” without further pre-specification [21, 25, 29–31, 33] but included details on specific symptoms in the result section, while three studies did not give any information on the planned outcome assessment [22, 26, 27]. Regarding reporting of this outcome, the latter three simply reported narratively that no adverse events or side effects had occurred.

### Life impact

Summarized in the core area “life impact”, we found reports of the related outcome domains “quality of life” (six studies [21, 23, 25–27, 30], 46%) and “physical functioning” (two studies [26, 27], 15%). Mean or median scores were reported and assessed with a wide variety of questionnaires and scales at diverging time points (see Table 4).

Thematically different, however, classified under “delivery of care” in the same core area, were study feasibility aspects, reported by three studies [25–27] (23%) as their primary outcome.

### Resource use

From the core area of “resource use,” there were three outcome domains reported by several studies described in the following (see Table 4).

### Need for further intervention

The outcome domain “need for further intervention”, more specifically the need for blood transfusions, was reported in three studies as a primary outcome and in seven studies as a secondary outcome. Specific outcome definitions varied from the number or percentage of patients receiving blood transfusion, number of blood units transfused, or amount of transfused blood in total or per patient in mean ± SD or median with interquartile range (IQR) to odds of needing blood transfusion. Variance in the timing of outcome measurement across studies was equally large ranging from 30 days preoperatively to 30 days postoperatively with many time points in between as well as less specified timeframes such as “until discharge” or “during hospital stay”.

### Hospital resources

The use of hospital resources was reported as the total length of hospital or intensive care unit (ICU) stay (mean (days) ± SD/median with IQR) or as the total number of patients needing ICU admission or readmission to the hospital after discharge within 30 days or not further specified timeframes. Overall, there were eight studies (62%) investigating this outcome domain as a secondary outcome.

### Economic

Among all 13 studies, there was only one study [32] (8%) comparing cost savings per patient between treatment groups from hospital admission to discharge.

**Fig. 2:**
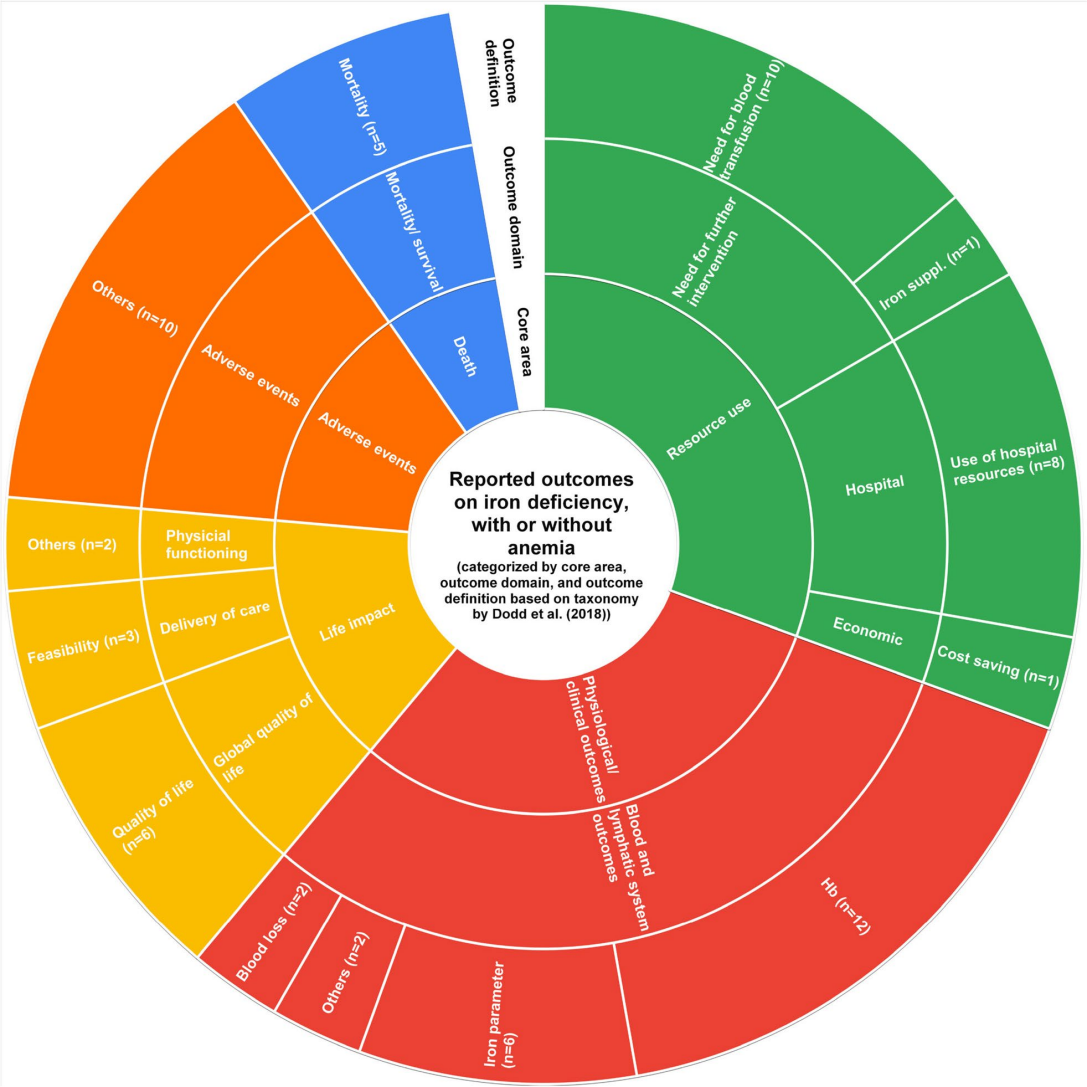
Overview of reported outcomes (based on outcome taxonomy by Dodd et al. [18])

A graphic overview of all reported outcomes categorized by core area and outcome domain is provided in Fig. 2.

A common combination of outcome measures was “Hb level” (core area: physiological/clinical outcomes; outcome domain: blood and lymphatic system outcomes) and “need for blood transfusion” (core area: resource use; outcome domain: need for further intervention), which was reported by *n*=8 studies [21, 24–27, 31–33]. Further, the combination of “need for blood transfusion” (core area: resource use; outcome domain: need for further intervention) and “use of hospital resources” (core area: resource use; outcome domain: hospital) was also assessed by almost the same *n*=8 studies [21, 25–29, 31, 32].

### Sample size calculations and clinically relevant effects

Eight studies (62%), of which two performed propensity-score matching [28, 33], provided a sample size calculation. Three studies estimated their sample size on the transfusion rate (core area, resource use; outcome domain, need for further intervention) as the primary outcome and aimed for a reduction of about 50% between groups (with an assumed raw transfusion rate between 30 and 45%) [21, 24, 28]. Three studies used changes in Hb levels (core area: physiological/clinical outcomes; outcome domain: blood and lymphatic system outcomes) for their sample size calculation: Two of these studies utilized an Hb difference of 1 g/dL with an estimated SD: 1.2 to 1.5 g/dL, as primary outcome [22, 27]. However, both studies differed in the Hb assessment time points: Kim et al. calculated the difference between preoperative and postoperative Hb levels but did not provide an exact definition of these time points. Klein et al. evaluated a Hb change from baseline to presurgery (i.e., day of surgery) (within 10 to 42 days according to trial registration) [27]. Scardino et al. stated a Hb reduction of 0.2 g/dL in the intervention group and of about 0.3 g/dL in the control group as a clinically relevant effect (estimated SD: 0.3 g/dL) [32]. Thin et al. calculated their sample size to show feasibility (core area, life impact; outcome domain, delivery of care), which was defined by at least 97% of participants receiving the drug within 5 days of enrollment [25].

### Methodological quality of outcomes

**Fig. 3:**
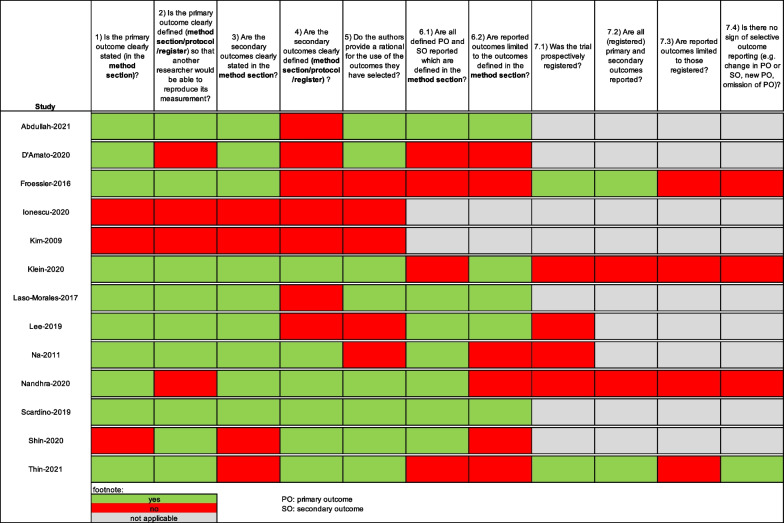
Quality assessment of reported outcomes

The methodological assessment of reported outcomes is presented in Fig. 3. Quality criteria regarding domains 1 to 6.2 and, therefore, applicable to RCTs and observational studies, were fulfilled (i.e., >50% of questions answered “yes”) by the majority of studies. Three domains that were most frequently not reached by studies were as follows: Seven studies (54%) did not clearly define the secondary outcomes (e.g., in the method section, protocol, or registry), six studies (46%) reported more outcomes than were defined in their method section, and five studies (39%) did not provide a rational for the utilized outcomes. Domains 7.1 to 7.4 regarding trial registration and selective outcome reporting were only assessed for prospective studies (*n*=6, Klein 2020 and Nandhra 2020 counted as one trial (i.e., CAVIAR study)). These domains were newly introduced by our approach and therefore not part of the MOMENT criteria [20]. Two (67%) of *n*=3 trials (Klein 2020 and Nandhra 2020 counted as one trial (i.e., CAVIAR study)) were registered prospectively (i.e., registration date before the first patient recruited [34]). Only one trial (17%) showed no sign of selective outcome reporting (e.g., change in primary outcome or secondary outcome, new primary outcome, omission of primary outcome).

## Discussion

To the best of our knowledge, this is the first systematic review that identified and appraised outcomes reported for preoperative or perioperative treatment of ID, with or without anemia, from 13 RCTs and observational studies in ID(A) confirmed patients in a non-perinatal setting. Comparability between studies investigating the same disease is necessary to generate reliable evidence on the respective condition’s treatment by calculating overall effect estimates in meta-analyses. In the context of ID research, studies lacking to define and asses ID in their anemic patients are not appropriate to investigate the efficacy of iron supplementation on ID(A), since different forms of anemia need different therapy approaches to be sure to treat the underlying cause. Therefore, studies that do not define ID(A) as inclusion criteria of the study population were not eligible for the current systematic review. Here, the development of a COS based on an established classification taxonomy proposed by Dodd et al. [18] plays a vital role in the harmonization of data with regard to ID(A) studies. In our systematic review, studies showed relative consistency regarding the usage of the WHO definition of anemia; however, high heterogeneity was observed regarding the diagnosis of ID as well as details on interventions (IV vs. oral iron, various preparations, etc.) and comparators (active comparators vs. SoC). An important finding that we had not planned to investigate but became apparent during our review was the studies’ failure to define and follow patients’ inclusion criteria regarding ID(A). We had to exclude twenty RCTs that investigated iron and/or erythropoietin supplementation in anemia but did not further diagnose the reason for anemia before enrolment of all study participants. Around one-third stated that they have treated IDA without verifying the diagnosis. Unnecessary supplementation of iron is critical since iron overload can cause harm in some cases (e.g., kidney damage). We decided to exclude those studies due to their low comparability with studies specifically focusing on laboratory-confirmed ID(A). Especially in terms of clinical trials, testing for efficacy of therapies—such as iron supplementation—the to-be-treated disease should be clearly defined and present in participants (i.e., confirmed diagnosis), though unnecessary or harmful treatment can be avoided and cause-effect relationships can be drawn (solely) between intervention and outcome. Therefore, researchers when planning future trials should consider proper definitions and follow in- and exclusion criteria. The current recommendations from the International Consensus Conference on Anemia Management in Surgical Patients (ICCAMS) state that an appropriate therapy for anemia should be guided by an accurate diagnosis of the etiology [35].

In our study pool, the most frequently investigated outcomes were related to the measurement of Hb levels (92% of studies), adverse events (77% of studies), the need for blood transfusion (77%) as well as the use of hospital resources (62%). Although this suggests conformity to some extent, measuring methods and time points varied widely and would lead to limited comparability when planning to perform a meta-analysis, ultimately lowering the quality of the evidence. There is still a need for a clearer definition and clinical reasoning of how and when those outcomes should be assessed in trials investigating the efficacy of intravenous iron supplementation in ID patients. Some outcomes were widely scattered including mortality, other blood outcomes such as IDA-related laboratory parameters or blood loss as well as a variety of outcomes in the core area of “life impact”. Except for mortality, the lack of detail on assessment methods was especially apparent at this point and limited reproducibility of the studies’ results.

Recent systematic reviews investigating patients undergoing preoperative treatment with iron monotherapy compared to placebo, SoC, or no intervention showed a risk reduction regarding allogenic blood transfusion [12–14], of which two meta-analyses did not reach statistical significance [13, 14] (e.g., optimal information size was not reached in review by Ng et al.). Across all outcomes, the reviews showed high levels of uncertainty. The aforementioned heterogeneity of included patients, amongst other reasons, limited the certainty of evidence. Elhenawy et al. included studies with all preoperative patients receiving iron supplementation whereas Ng et al. and Van Remoortel solely included anemic patients irrespective of their etiology [12–14]. The efficacy of iron plus erythropoietin in non-cardiac surgery patients was investigated in a systematic review by Kaufner et al. [36]. The authors found that erythropoietin plus iron can reduce the need for blood transfusions, and if administered in high doses, the combined intervention can increase preoperative Hb levels. Nevertheless, a confirmed ID was not an inclusion criterion for RCTs by Kaufner et al. [36]. In addition to the heterogeneities with regard to anemia etiology in included patients, as depicted by the systematic reviews mentioned above, our systematic review highlights the great heterogeneity of reported outcomes across ID(A) trials, which might constitute another reason for hampered evidence synthesis since consistent time points of measures and clear outcomes are lacking. Furthermore, future RCTs need sufficiently powered sample sizes, participants with defined anemia conditions (e.g., ID in case of iron treatment) as well as a consented COS.

The main strength of our review is the thorough systematic search for clinical trials as well as observational studies in the field of ID(A). Thus, allowing a comprehensive summary of reported outcomes measuring the efficacy and effectiveness of iron interventions is possible. All of these eligible studies provided a confirmed diagnosis of ID(A) to guarantee iron supplementation treats the underlying cause of anemia. Studies without analysis restricted to ID(A) patients solely (e.g., Triphaus et al. [37]) or with suspected (i.e., not laboratory confirmed) ID(A) (e.g., Richards et al. [38]) were therefore excluded from our systematic review. Furthermore, our strict approach with the exclusion of studies without confirmed ID guarantees that meaningful outcomes regarding iron status and ID (e.g., ferritin or TSAT) are utilized for our summarization of existing evidence. Outcomes not suitable in the context of ID(A) were avoided by our approach. The identification of reported outcomes is the first step in the development of a COS and further outcomes, which might not have been reported by the identified studies can still be proposed by experts (e.g., trialists) in the consensus conference. This combined approach in developing a COS as proposed by the COMET initiative accounts in addition to reporting bias. Second, outcomes utilized in clinical trials and observational studies were not only extracted throughout the published manuscript. This thorough approach allowed for identifying outcomes not reported in publications and portraying a better view of important outcomes for ID(A) studies. Third, our summary of outcomes was based on the classification taxonomy system proposed by Dodd et al. [18]. Outcome taxonomy improves the consistency of outcome classification between trials as a main goal of COS development. Furthermore, future research benefits from this data harmonization in terms of searching (e.g., throughout the COS database of COMET) and outcome assessment (especially for meta-analysis) [18]. Fourth, the quality of outcome definition and reporting was assessed using the MOMENT criteria [20]. The MOMENT criteria comprise questions in terms of outcome definition, rationale for outcomes, and quality of measurement and were also utilized in former systematic reviews on COS development [39–41]. Fifth, clinically relevant effects stated in publications for sample size calculation were extracted and summarized. Thus, informed discussion on clinically relevant differences (e.g., by DELPHI group on COS development) is possible and might inform sample size calculation of future studies.

However, there are also limitations. Although our COS represents a comprehensive picture of outcomes assessed in clinical trials and observational studies with ID(A) patients undergoing iron supplementation, our findings do not address how relevant these outcomes are for clinicians, patients, and policymakers. This was not the aim of this systematic review and will be undertaken in the next step as described in the COMET Handbook on COS development [15]. Only studies from 2000 to April 1, 2022, were included to summarize studies representing the latest research on iron supplementation in ID(A) patients. Appraisal of study quality (e.g., using Cochrane risk of bias (RoB) 2) was not carried out. However, the main scope of this review was to systematically identify and assess reported outcomes. Effect sizes reported were not of interest, and therefore, no bias regarding study quality or missing data on outcomes was considered. Nevertheless, domains like selective reporting, which is also included in Cochrane RoB 2, were added to our critical appraisal.

## Conclusions

Despite the high prevalence of ID and IDA in the preoperative setting, there is still no consent for an adequate treatment plan in place. Due to the described heterogeneities regarding outcome reporting, reliable evidence of the efficacy and safety of iron supplementation (e.g., by meta-analyses) is lacking. This review poses the first step for developing a COS in the field of preoperative correction of ID(A). Subsequently, the relevancy of the collected outcomes has to be evaluated in a DELPHI process by clinicians, patients, and stakeholders, considering health, quality of life, and resources being used. Our ultimate goal is to provide a thoroughly scrutinized COS, agreed on by a consensus conference, to guide future trials and to inform quality improvement initiatives.

### Supplementary information

**Additional file 1.** Search strategy – MEDLINE, CENTRAL, and clinicaltrials.gov

**Additional file 2.** Study characteristics of excluded randomized controlled trials due to insufficient in-/exclusion criteria

**Additional file 3.** Extracted variables

**Additional file 4.** Detailed study characteristics of eligible studies on iron deficiency, with or without anemia

## Data Availability

Extracted data can be retrieved on reasonable request from the corresponding author.

## Abbreviations

CENTRAL: Cochrane Central Register of Controlled Trials
COMET: Core Outcome Measures in Effectiveness Trials
COS: Core outcome set
CRP: C-reactive protein
ESC: European Society of Cardiology
Hb: Hemoglobin
ICCAMS: International Consensus Conference on Anemia Management in Surgical Patients
ICU: Intensive care unit
ID: Iron deficiency
IDA: Iron deficiency anemia
ID(A): Iron deficiency with or without anemia
IQR: Interquartile range
IV: Intravenous
MeSH: Medical subject headings
PBM: Patient Blood Management
PRISMA: Preferred Reporting Items for Systematic Reviews and Meta-Analyses
RCT: Randomized controlled trial
RoB: Risk of bias
SD: Standard deviation
SoC: Standard of care
WHO: World Health Organization
