# Supplementary material for: Reported outcomes in patients with iron deficiency or iron deficiency anemia undergoing major surgery: a systematic review of outcomes
Source: Syst Rev. 2024 Jan 2;13:5. doi: 10.1186/s13643-023-02431-x (PMC10759584; doi:10.1186/s13643-023-02431-x)
Supplement: Supplementary file 1 — Additional file 1. Search strategy – MEDLINE, CENTRAL, and clinicaltrials.gov [file 13643_2023_2431_MOESM1_ESM.docx]

**Reported outcomes in patients with iron deficiency or iron deficiency anemia undergoing major surgery: a systematic review of outcomes**

**Additional file 1. Search strategy – MEDLINE, CENTRAL, and clinicaltrials.gov**

MEDLINE (via Pubmed):

Search Date: 01/04/2022

| Search | Query | Results |
| --- | --- | --- |
| #5 | Search: #1 AND #2 AND #3 AND #4 Filters: from 2000/1/1 - 2022/4/1 | [1,858](https://pubmed.ncbi.nlm.nih.gov/?term=%231+AND+%232+AND+%233+AND+%234+&sort=&filter=dates.2000%2F1%2F1-2022%2F4%2F1) |
| #4 | Search: (clinical trial[pt] OR comparative study[pt] OR (control[tw] AND study[tw]) OR program[tw] OR epidemiologic studies[mh]) NOT ((animals[mh:noexp] NOT humans[mh:noexp]) OR comment[pt] OR editorial[pt] OR review[pt] OR meta analysis[pt] OR case report[tw] OR consensus[mh] OR guideline[pt] OR history[sh]) Filters: from 2000/1/1 - 2022/4/1 | [3,943,419](https://pubmed.ncbi.nlm.nih.gov/?term=%28clinical+trial%5Bpt%5D+OR+comparative+study%5Bpt%5D+OR+%28control%5Btw%5D+AND+study%5Btw%5D%29+OR+program%5Btw%5D+OR+epidemiologic+studies%5Bmh%5D%29+NOT+%28%28animals%5Bmh%3Anoexp%5D+NOT+humans%5Bmh%3Anoexp%5D%29+OR+comment%5Bpt%5D+OR+editorial%5Bpt%5D+OR+review%5Bpt%5D+OR+meta+analysis%5Bpt%5D+OR+case+report%5Btw%5D+OR+consensus%5Bmh%5D+OR+guideline%5Bpt%5D+OR+history%5Bsh%5D%29+&sort=&filter=dates.2000%2F1%2F1-2022%2F4%2F1) |
| #3 | Search: (((((((Iron[MeSH Terms]) OR (Iron[Title/Abstract])) OR (ferritins[MeSH Terms])) OR (ferr*[Title/Abstract])) OR (erythropoietin[MeSH Terms])) OR (erythropo*[Title/Abstract]))) OR (Darbepoetin[Title/Abstract]) Filters: from 2000/1/1 - 2022/4/1 | [256,968](https://pubmed.ncbi.nlm.nih.gov/?term=%28%28%28%28%28%28%28Iron%5BMeSH+Terms%5D%29+OR+%28Iron%5BTitle%2FAbstract%5D%29%29+OR+%28ferritins%5BMeSH+Terms%5D%29%29+OR+%28ferr%2A%5BTitle%2FAbstract%5D%29%29+OR+%28erythropoietin%5BMeSH+Terms%5D%29%29+OR+%28erythropo%2A%5BTitle%2FAbstract%5D%29%29%29+OR+%28Darbepoetin%5BTitle%2FAbstract%5D%29&sort=&filter=dates.2000%2F1%2F1-2022%2F4%2F1) |
| #2 | Search: (((((((perioperative care[MeSH Terms]) OR (perioperative period[MeSH Terms])) OR (perioperative[Title/Abstract])) OR (peri-operative[Title/Abstract])) OR (preoperative[Title/Abstract])) OR (pre-operative[Title/Abstract])) OR (surg*[Title/Abstract])) OR (surgical procedures, operative[MeSH Terms]) Filters: from 2000/1/1 - 2022/4/1 | [2,880,532](https://pubmed.ncbi.nlm.nih.gov/?term=%28%28%28%28%28%28%28perioperative+care%5BMeSH+Terms%5D%29+OR+%28perioperative+period%5BMeSH+Terms%5D%29%29+OR+%28perioperative%5BTitle%2FAbstract%5D%29%29+OR+%28peri-operative%5BTitle%2FAbstract%5D%29%29+OR+%28preoperative%5BTitle%2FAbstract%5D%29%29+OR+%28pre-operative%5BTitle%2FAbstract%5D%29%29+OR+%28surg%2A%5BTitle%2FAbstract%5D%29%29+OR+%28surgical+procedures%2C+operative%5BMeSH+Terms%5D%29+&sort=&filter=dates.2000%2F1%2F1-2022%2F4%2F1) |
| #1 | Search: ((((((anemia[MeSH Terms]) OR (anemi*[Title/Abstract])) OR (anaemi*[Title/Abstract])) OR (hemoglobins[MeSH Terms])) OR (hemoglobin*[Title/Abstract])) OR (haemoglobin*[Title/Abstract])) OR (anemia, iron deficiency[MeSH Terms]) Filters: from 2000/1/1 - 2022/4/1 | [254,219](https://pubmed.ncbi.nlm.nih.gov/?term=%28%28%28%28%28%28anemia%5BMeSH+Terms%5D%29+OR+%28anemi%2A%5BTitle%2FAbstract%5D%29%29+OR+%28anaemi%2A%5BTitle%2FAbstract%5D%29%29+OR+%28hemoglobins%5BMeSH+Terms%5D%29%29+OR+%28hemoglobin%2A%5BTitle%2FAbstract%5D%29%29+OR+%28haemoglobin%2A%5BTitle%2FAbstract%5D%29%29+OR+%28anemia%2C+iron+deficiency%5BMeSH+Terms%5D%29+&sort=&filter=dates.2000%2F1%2F1-2022%2F4%2F1) |

Cochrane Centre Register of Controlled Trials (CENTRAL) (via Cochrane Library):

Search Name: COS-IDA
Search Date: 01/04//2022

**ID** Search

**#1** MeSH descriptor: [Anemia] explode all trees

**#2** (anemi*):ti,ab,kw with Cochrane Library publication date Between Jan 2000 and Apr 2022

**#3** (anaemi*):ti,ab,kw with Cochrane Library publication date Between Jan 2000 and Apr 2022

**#4** MeSH descriptor: [Hemoglobins] explode all trees

**#5** (hemoglobin*):ti,ab,kw with Cochrane Library publication date Between Jan 2000 and Apr 2022

**#6** (haemoglobin*):ti,ab,kw with Cochrane Library publication date Between Jan 2000 and Apr 2022

**#7** MeSH descriptor: [Anemia, Iron-Deficiency] explode all trees

**#8** MeSH descriptor: [Perioperative Care] explode all trees

**#9** MeSH descriptor: [Perioperative Period] explode all trees

**#10** (preoperative):ti,ab,kw with Cochrane Library publication date Between Jan 2000 and Apr 2022

**#11** (pre-operative):ti,ab,kw with Cochrane Library publication date Between Jan 2000 and Apr 2022

**#12** (perioperative):ti,ab,kw with Cochrane Library publication date Between Jan 2000 and Apr 2022

**#13** (peri-operative):ti,ab,kw with Cochrane Library publication date Between Jan 2000 and Apr 2022

**#14** (surg*):ti,ab,kw with Cochrane Library publication date Between Jan 2000 and Apr 2022

**#15** MeSH descriptor: [Surgical Procedures, Operative] explode all trees

**#16** MeSH descriptor: [Iron] explode all trees

**#17** (iron):ti,ab,kw with Cochrane Library publication date Between Jan 2000 and Apr 2022

**#18** MeSH descriptor: [Ferritins] explode all trees

**#19** (Ferr*):ti,ab,kw with Cochrane Library publication date Between Jan 2000 and Jan 2022

**#20** MeSH descriptor: [Erythropoietin] explode all trees

**#21** (erythropo*):ti,ab,kw with Cochrane Library publication date Between Jan 2000 and Apr 2022

**#22** ("darbepoetin"):ti,ab,kw with Cochrane Library publication date Between Jan 2000 and Apr 2022

**#23** (#1 OR #2 OR #3 OR #4 OR #5 OR #6 OR #7) AND (#8 OR #9 OR #10 OR #11 OR #12 OR #13 OR #14 OR #15) AND (#16 OR #17 OR #18 OR #19 OR #20 OR #21 OR #22) with Cochrane Library publication date Between Jan 2000 and Apr 2022

clinicaltrials.gov:

Completed Studies | Interventional Studies | Iron Deficiency anemia | OR iron-deficiency OR anemia OR anaemia | Darbepoetin OR Ferric OR Iron OR Erythropoetin | Adult, Older Adult | Results first posted from 01/01/2000 to 01/04/2022

**References**

1. Biboulet P, Bringuier S, Smilevitch P, Loupec T, Thuile C, Pencole M, et al. Preoperative Epoetin-α with Intravenous or Oral Iron for Major Orthopedic Surgery: A Randomized Controlled Trial. Anesthesiology. 2018;129(4):710-20.

2. Bielza R, Llorente J, Thuissard IJ, Andreu-Vázquez C, Blanco D, Sanjurjo J, et al. Effect of intravenous iron on functional outcomes in hip fracture: a randomised controlled trial. Age Ageing. 2021;50(1):127-34.

3. Buljan M, Nemet D, Golubic-Cepulic B, Bicanic G, Tripkovic B, Delimar D. Two different dosing regimens of human recombinant erythropoietin beta during preoperative autologous blood donation in patients having hip arthroplasty. International Orthopaedics. 2012;36(4):703-9.

4. Christodoulakis M, Tsiftsis DD. Preoperative Epoetin Alfa in Colorectal Surgery: A Randomized, Controlled Study. Annals of Surgical Oncology. 2005;12(9):718-25.

5. Dousias V, Paraskevaidis E, Dalkalitsis N, Tsanadis G, Navrozoglou I, Lolis D. Recombinant human erythropoietin in mildly anemic women before total hysterectomy. Clin Exp Obstet Gynecol. 2003;30(4):235-8.

6. Edwards TJ, Noble EJ, Durran A, Mellor N, Hosie KB. Randomized clinical trial of preoperative intravenous iron sucrose to reduce blood transfusion in anaemic patients after colorectal cancer surgery. Br J Surg. 2009;96(10):1122-8.

7. Kateros K, Sakellariou VI, Sofianos IP, Papagelopoulos PJ. Epoetin alfa reduces blood transfusion requirements in patients with intertrochanteric fracture. Journal of Critical Care. 2010;25(2):348-53.

8. Keeler BD, Simpson JA, Ng O, Padmanabhan H, Brookes MJ, Acheson AG. Randomized clinical trial of preoperative oral versus intravenous iron in anaemic patients with colorectal cancer. Br J Surg. 2017;104(3):214-21.

9. Kosmadakis N, Messaris E, Maris A, Katsaragakis S, Leandros E, Konstadoulakis MM, et al. Perioperative Erythropoietin Administration in Patients With Gastrointestinal Tract Cancer: Prospective Randomized Double-Blind Study. Annals of Surgery. 2003;237(3):417-21.

10. Larson B, Bremme K, Clyne N, Nordström L. Preoperative treatment of anemic women with epoetin beta. Acta Obstet Gynecol Scand. 2001;80(6):559-62.

11. Lidder PG, Sanders G, Whitehead E, Douie WJ, Mellor N, Lewis SJ, et al. Pre-operative oral iron supplementation reduces blood transfusion in colorectal surgery - a prospective, randomised, controlled trial. Ann R Coll Surg Engl. 2007;89(4):418-21.

12. M. MAFC, W. TBJ, T. KN, J. vOJ, D. VA, P. P. Pre-operative injections of epoetin-α versus post-operative retransfusion of autologous shed blood in total hip and knee replacement. The Journal of Bone and Joint Surgery British volume. 2008;90-B(8):1079-83.

13. Phipps O, Al-Hassi HO, Quraishi MN, Dickson EA, Segal J, Steed H, et al. Oral and Intravenous Iron Therapy Differentially Alter the On- and Off-Tumor Microbiota in Anemic Colorectal Cancer Patients. Cancers (Basel). 2021;13(6).

14. Richards T, Baikady RR, Clevenger B, Butcher A, Abeysiri S, Chau M, et al. Preoperative intravenous iron to treat anaemia before major abdominal surgery (PREVENTT): a randomised, double-blind, controlled trial. Lancet. 2020;396(10259):1353-61.

15. Rosencher N, Poisson D, Albi A, Aperce M, Barré J, Samama CM. Two injections of erythropoietin correct moderate anemia in most patients awaiting orthopedic surgery. Canadian Journal of Anesthesia. 2005;52(2):160-5.

16. Scott SN, Boeve TJ, McCulloch TM, Fitzpatrick KA, Karnell LH. The Effects of Epoetin Alfa on Transfusion Requirements in Head and Neck Cancer Patients: A Prospective, Randomized, Placebo-Controlled Study. The Laryngoscope. 2002;112(7):1221-9.

17. TAHIR S, SAEED K, NAZEER S. Comparison of Intravenous Iron Sucrose alone Versus Intravenous Iron Sucrose Along With Erythropoietin for Management of Anemia for Gynecological Patients Waiting for Surgery.

18. Marina U, Maria Del T, Omar Abdul-Jawad A, Francisco C-P, Ander R, Robert De L, et al. Combined erythropoietin and iron therapy for anaemic patients undergoing transcatheter aortic valve implantation: the EPICURE randomised clinical trial. EuroIntervention. 2017;13(1):44-52.

19. Weber EWG, Slappendel R, Hémon Y, Mähler S, Dalén T, Rouwet E, et al. Effects of epoetin alfa on blood transfusions and postoperative recovery in orthopaedic surgery: the European Epoetin Alfa Surgery Trial (EEST). European Journal of Anaesthesiology | EJA. 2005;22(4):249-57.

20. Abdullah HR, Thamnachit T, Hao Y, Lim WY, Teo LM, Sim YE. Real-world results of the implementation of preoperative anaemia clinic with intravenous iron therapy for treating iron-deficiency anaemia: a propensity-matched case-control study. Annals of Translational Medicine. 2020;9(1):6.

21. D'Amato T, Kon E, Martorelli F, Monteleone G, Simili V, Tasso F, et al. Effect of intravenous ferric carboxymaltose supplementation in non-anaemic iron deficient patients undergoing hip and knee arthroplasty. J Biol Regul Homeost Agents. 2020;34(4 Suppl. 3):69-77. Congress of the Italian Orthopaedic Research Society.

22. Froessler B, Palm P, Weber I, Hodyl NA, Singh R, Murphy EM. The Important Role for Intravenous Iron in Perioperative Patient Blood Management in Major Abdominal Surgery: A Randomized Controlled Trial. Annals of Surgery. 2016;264(1).

23. Ionescu A, Sharma A, Kundnani NR, Mihăilescu A, David VL, Bedreag O, et al. Intravenous iron infusion as an alternative to minimize blood transfusion in peri-operative patients. Scientific Reports. 2020;10(1):18403.

24. Kim YH, Chung HH, Kang SB, Kim SC, Kim YT. Safety and Usefulness of Intravenous Iron Sucrose in the Management of Preoperative Anemia in Patients with Menorrhagia: A Phase IV, Open-Label, Prospective, Randomized Study. Acta Haematologica. 2009;121(1):37-41.

25. Klein AA, Chau M, Yeates JA, Collier T, Evans C, Agarwal S, et al. Preoperative intravenous iron before cardiac surgery: a prospective multicentre feasibility study. British Journal of Anaesthesia. 2020;124(3):243-50.

26. Laso-Morales M, Jericó C, Gómez-Ramírez S, Castellví J, Viso L, Roig-Martínez I, et al. Preoperative management of colorectal cancer–induced iron deficiency anemia in clinical practice: data from a large observational cohort. Transfusion. 2017;57(12):3040-8.

27. Lee S, Ryu K-J, Lee ES, Lee KH, Lee JJ, Kim T. Comparative efficacy and safety of intravenous ferric carboxymaltose and iron sucrose for the treatment of preoperative anemia in patients with menorrhagia: An open-label, multicenter, randomized study. Journal of Obstetrics and Gynaecology Research. 2019;45(4):858-64.

28. Na H-S, Shin S-Y, Hwang J-Y, Jeon Y-T, Kim C-S, Do S-H. Effects of intravenous iron combined with low-dose recombinant human erythropoietin on transfusion requirements in iron-deficient patients undergoing bilateral total knee replacement arthroplasty (CME). Transfusion. 2011;51(1):118-24.

29. Nandhra S, Chau M, Klein AA, Yeates JA, Collier T, Evans C, et al. Preoperative anaemia management in patients undergoing vascular surgery. Br J Surg. 2020;107(12):1558-61.

30. Scardino M, Di Matteo B, Martorelli F, Tanzi D, Kon E, D’Amato T. Improved patient blood management and cost saving in hip replacement surgery through the implementation of pre-operative Sucrosomial® iron supplementation: a quality improvement assessment study. International Orthopaedics. 2019;43(1):39-46.

31. Shin K-H, Park J-H, Jang K-M, Hong S-H, Han S-B. Effects of intravenous iron monotherapy for patients with iron deficient anemia undergoing total knee arthroplasty. Arthroplasty. 2020;2(1):22.

32. Thin TN, Tan BPY, Sim EY, Shum KL, Chan HSP, Abdullah HR. Preoperative Single-Dose Intravenous Iron Formulation to Reduce Postsurgical Complications in Patients Undergoing Major Abdominal Surgery: A Randomized Control Trial Feasibility Study (PIRCAS Trial Pilot). Cureus. 2021;13(8):e17357.
