# Supplementary material for: Reported outcomes in patients with iron deficiency or iron deficiency anemia undergoing major surgery: a systematic review of outcomes
Source: Syst Rev. 2024 Jan 2;13:5. doi: 10.1186/s13643-023-02431-x (PMC10759584; doi:10.1186/s13643-023-02431-x)
Supplement: Supplementary file 2 — Additional file 2. Study characteristics of excluded randomized controlled trials due to insufficient in-/exclusion criteria [file 13643_2023_2431_MOESM2_ESM.docx]

**Reported outcomes in patients with iron deficiency or iron deficiency anemia undergoing major surgery: a systematic review of outcomes**

**Additional file 2. Study characteristics of excluded randomized controlled trials due to insufficient in-/exclusion criteria**

| **Study ID** | **Rationale/aim/idea of the study, cited from study** | **Investigated population according to study** | **Type of surgery** | **Definition of anemia as inclusion criterion by study** | **Definition of iron deficiency as inclusion criterion by study** | **Inter-vention** | **Compa-rator** | **Primary outcome according to study** | **Assessment** |
| --- | --- | --- | --- | --- | --- | --- | --- | --- | --- |
| Biboulet 2018  (1) | "The aim of this study was to compare the clinical effects of erythropoietin in combination with oral or intravenous iron supplementation" | Pts. scheduled for elective hip or knee arthroplasty with Hb 10-13 g/dl | Orthopaedic surgery | Hb 10-13 mg/dl | NA | EPO + Iron IV | EPO + Iron oral | Change in Hb | Predefined aim was to investigate anaemia in general; respective inclusion criterion provided; no focus on IDA |
| Bielza 2021  (2) | "To determine the safety and effect of intravenous iron sucrose on functional outcomes, delirium, nosocomial infections, and transfusion requirements in older patients with hip fracture." | Pts. 70 years and older undergoing hip fracture surgery | Orthopaedic surgery | NA | NA | Iron IV | Placebo | Absolute function-al gain score | There is no explicit claim that pts. with iron deficiency anaemia were examined. However, the study’s introduction implies focus on iron deficiency anaemia without defining it as an inclusion criterion. |

| *Additional file 2.continued* | | | | | | | | | |
| --- | --- | --- | --- | --- | --- | --- | --- | --- | --- |
| **Study ID** | **Rationale/aim/idea of the study, cited from study** | **Investigated population according to study** | **Type of surgery** | **Definition of anemia as inclusion criterion by study** | **Definition of iron deficiency as inclusion criterion by study** | **Inter-vention** | **Compa-rator** | **Primary outcome according to study** | **Assessment** |
| Buljan 2012  (3) | "Our aim was to evaluate the effectiveness of two different dosing regimens of human recombinant erythropoietin (rHu-EPO) for preoperative autologous blood collection in patients undergoing total hip arthroplasty (THA)." | Pts. 60-80 years scheduled for primary THA for osteoarthritis and able to donate autologous blood preoperatively | Orthopaedic surgery | Hb 10.5-13 mg/dl | NA | EPO + Iron oral | Iron oral | Efficacy of intervention to keep Hb high enough before surgery to donate two units of autolo-gous blood and transfusion rate | Predefined aim was to investigate anaemia in general; respective inclusion criterion provided; no focus on IDA |
| Christo-doulakis 2005  (4) | "We investigated the possible benefits of perioperative epoetin alfa administration in anaemic patients to correct haemoglobin levels and reduce transfusion needs." | Pts. aged >18 years undergoing elective colorectal surgery for respectable colorectal cancer in 18 centres in Greece | Colorectal surgery | Hb 9-12 mg/dl | NA | EPO + Iron oral | Iron oral | Transfusion rate | Predefined aim was to investigate anaemia in general; respective inclusion criterion provided; no focus on IDA |

| *Additional file 2.continued* | | | | | | | | | |
| --- | --- | --- | --- | --- | --- | --- | --- | --- | --- |
| **Study ID** | **Rationale/aim/idea of the study, cited from study** | **Investigated population according to study** | **Type of surgery** | **Definition of anemia as inclusion criterion by study** | **Definition of iron deficiency as inclusion criterion by study** | **Inter-vention** | **Compa-rator** | **Primary outcome according to study** | **Assessment** |
| Dousias 2003  (5) | “The purpose […] was to investigate the efficacy of preoperative recombinant human erythropoietin treatment in a group of mildly anaemic women” | Mildly anaemic women who underwent total hysterectomy for leiomyomas | Gynaecological surgery | Hb 9-12 mg/dl | In contrary, the inclusion criterion was ferritin > 50ng/ml | EPO + Iron | Iron | Transfu-sion rate | Predefined aim was to investigate anaemia in general; respective inclusion criterion provided; no focus on IDA |
| Edwards 2009  (6) | “This study was designed to determine whether iron sucrose reduces the likelihood of postoperative blood transfusion in patients undergoing elective colorectal cancer resection.” | Pts. undergoing  resectional surgery with a preoperative diagnosis of colorectal cancer" | Colorectal surgery | NA | NA | Iron IV | Placebo | Change in Hb; transfusion rate | There is no explicit claim that pts. with iron deficiency were examined. However, the study’s introduction implies focus on iron deficiency without defining it as an inclusion criterion |
| Kateros 2010  (7) | “The purpose of this study is to identify the potential benefits or complications from the use of epoetin alfa in patients with intertrochanteric fracture.” | Pts. who sustained an intertrochanteric fracture | Orthopaedic surgery | Not as inclusion criterion, but exclusion criterion:  Hb >13 mg/dl | NA | EPO + Iron IV | Placebo + Iron IV | Transfusion rate | Predefined aim was to investigate anaemia in general; respective inclusion criterion provided; no focus on IDA |

| *Additional file 2.continued* | | | | | | | | | |
| --- | --- | --- | --- | --- | --- | --- | --- | --- | --- |
| **Study ID** | **Rationale/aim/idea of the study, cited from study** | **Investigated population according to study** | **Type of surgery** | **Definition of anemia as inclusion criterion by study** | **Definition of iron deficiency as inclusion criterion by study** | **Inter-vention** | **Compa-rator** | **Primary outcome according to study** | **Assessment** |
| Keeler 2017 (IVICA Trial)  (8) | “The study aimed to compare the efficacy of preoperative intravenous and oral iron in reducing blood transfusion use in anaemic patients undergoing elective colorectal cancer surgery.” | Anaemic pts. with non-metastatic colorectal adenocarcinoma undergoing elective colorectal cancer surgery | Colorectal surgery | Hb <12 (f) or  13 (m) g/dl | NA | Iron IV | Iron oral | Transfusion rate (first publication), Quality of life (second publication); 5-year survival rate (third publication) | There is explicit claim that the study mainly included IDA pts due to the reason that iron deficiency is the leading cause for anaemia in cancer patients. However, the study lacks defining a respective diagnostic inclusion criterion |
| Kosma-dakis 2003  (9) | "To investigate the effect of recombinant human erythropoietin (r-HuEPO) administration on perioperative haemoglobin concentrations and on the number of blood transfusions in patients undergoing surgery for gastrointestinal tract malignancies." | Diagnosis of nonmetastatic gastrointestinal malignancy, age 40 - 90 years, moderate anaemia | Gastrointestinal surgery | Hb 8.5-13 mg/dl | NA | EPO + Iron IV | Placebo + Iron IV | Transfusion rate | Predefined aim was to investigate anaemia in general; respective inclusion criterion provided; no focus on IDA |

| *Additional file 2.continued* | | | | | | | | | |
| --- | --- | --- | --- | --- | --- | --- | --- | --- | --- |
| **Study ID** | **Rationale/aim/idea of the study, cited from study** | **Investigated population according to study** | **Type of surgery** | **Definition of anemia as inclusion criterion by study** | **Definition of iron deficiency as inclusion criterion by study** | **Inter-vention** | **Compa-rator** | **Primary outcome according to study** | **Assessment** |
| Larson 2001  (10) | "To compare the effects of preoperative treatment with recombinant human erythropoietin (EPO) and iron with iron only on haemoglobin levels (Hb) in anaemic women prior to hysterectomy" | Otherwise healthy women with Hb less than 12 g/dL | Gynaecological surgery | Hb <12g/dl | NA | EPO + Iron oral | Iron oral | Change in Hb | Predefined aim was to investigate anaemia in general; respective inclusion criterion provided; no focus on IDA |
| Lidder 2007  (11) | "The aim of this study was to assess whether pre-operative oral iron therapy would decrease pre-operative anaemia and, thereby, reduce the incidence of peri-operative transfusion." | Pts. diagnosed with colorectal cancer and fit for surgery | Colorectal surgery | NA | NA | Iron oral | No treatment | Transfusion rate | There is an explicit claim that the study’s purpose is investigating pre-operatively anaemic pts. without defining anaemia as an inclusion criterion. |

| *Additional file 2. continued* | | | | | | | | | |
| --- | --- | --- | --- | --- | --- | --- | --- | --- | --- |
| **Study ID** | **Rationale/aim/idea of the study, cited from study** | **Investigated population according to study** | **Type of surgery** | **Definition of anemia as inclusion criterion by study** | **Definition of iron deficiency as inclusion criterion by study** | **Inter-vention** | **Compa-rator** | **Primary outcome according to study** | **Assessment** |
| Moonen 2008  (12) | "This prospective randomised clinical trial evaluated the effect of alternatives for allogeneic blood transfusions after total hip replacement and total knee replacement in patients with pre-operative haemoglobin levels between 10.0 g/dl and 13.0 g/dl." | Pts scheduled for elective total hip replacement or total knee replacement for primary osteoarthritis with a preoperative Hb level between 10.0 g/dl and 13.0 g/dl" | Orthopaedic surgery | Hb 10-13 mg/dl | NA | EPO + Iron IV | Auto-logous transfusion | Transfusion rate | Predefined aim was to investigate anaemia in general; respective inclusion criterion provided; no focus on IDA |
| Padmana-bhan 2019  (8) | "The present study aimed to compare the efficacy of preoperative intravenous ferric carboxymaltose (FCM) (Ferinject; Vifor Pharma, Surrey, UK) therapy with that of oral iron regarding haemoglobin levels and allogeneic red blood cell transfusion requirements for patients undergoing elective cardiac surgery." | Pts. scheduled for elective cardiac surgery, defined as coronary artery bypass graft and/or open valve surgery | Cardiac surgery | Hb <12 (f) or  13 (m) g/dl | NA | Iron IV | Iron oral | Change in Hb | Predefined aim was to investigate anaemia in general; respective inclusion criterion provided; no focus on IDA |

| *Additional file 2. continued* | | | | | | | | | |
| --- | --- | --- | --- | --- | --- | --- | --- | --- | --- |
| **Study ID** | **Rationale/aim/idea of the study, cited from study** | **Investigated population according to study** | **Type of surgery** | **Definition of anemia as inclusion criterion by study** | **Definition of iron deficiency as inclusion criterion by study** | **Inter-vention** | **Compa-rator** | **Primary outcome according to study** | **Assessment** |
| Phipps 2021  (13) | "This pilot intervention study aimed to compare the outcomes of oral and intravenous iron therapy on the on- and off-tumor microbiota, in order to assess which therapy is more beneficial to treat anaemia in iron-deficient colorectal cancer patients." | Anaemic pts. with non-metastatic colorectal adenocarcinoma undergoing elective colorectal cancer surgery (cohort of pts. from IVICA trial) | Colorectal surgery | Hb <12 (f) or  13 (m) g/dl | NA | Iron IV | Iron oral | On- and Off-Tumor Bacterial Diversity | There is explicit claim that the study mainly included iron deficient anaemic pts due to the reason that iron deficiency is the leading cause for anaemia in cancer patients. However, the study lacks defining a respective diagnostic inclusion criterion |

| *Additional file 2. continued* | | | | | | | | | |
| --- | --- | --- | --- | --- | --- | --- | --- | --- | --- |
| **Study ID** | **Rationale/aim/idea of the study, cited from study** | **Investigated population according to study** | **Type of surgery** | **Definition of anemia as inclusion criterion by study** | **Definition of iron deficiency as inclusion criterion by study** | **Inter-vention** | **Compa-rator** | **Primary outcome according to study** | **Assessment** |
| Richards 2021 (PREVENTT Trial)  (14) | “We aimed to test the hypothesis that intravenous iron given to anaemic patients before major open elective abdominal surgery would correct anaemia, reduce the need for blood transfusions, and improve patient outcomes.” | Adult pts. identified with anaemia at preoperative hospital visits before elective major open abdominal surgery | Major abdominal surgery (> 1h, non-laparoscopic) | Hb <12 (f) or  13 (m) g/dl | NA | Iron IV | Placebo | Composite endpoint of blood transfusion or death and the number of blood transfusion episodes from randomisation until 30 days after the index operation | There is no explicit claim that pts. with iron deficiency anaemia were examined. However, the study’s introduction implies focus on iron deficiency anaemia without defining it as an inclusion criterion. |

| *Additional file 2. continued* | | | | | | | | | |
| --- | --- | --- | --- | --- | --- | --- | --- | --- | --- |
| **Study ID** | **Rationale/aim/idea of the study, cited from study** | **Investigated population according to study** | **Type of surgery** | **Definition of anemia as inclusion criterion by study** | **Definition of iron deficiency as inclusion criterion by study** | **Inter-vention** | **Compa-rator** | **Primary outcome according to study** | **Assessment** |
| Rosen-cher 2005  (15) | "The primary objective of this study was to assess the number of erythropoietin (EPO) injections required to reach a haematocrit of 40% in moderately anaemic patients." | Pts. whose haematocrit concentrations were between 30 and 39% were enrolled 30 ± 9 days before the scheduled intervention | Orthopaedic surgery | Haematocrit value 30 to 39% | NA | EPO + Iron oral | Autologous transfusion + Iron oral | Change in haematocrit | Predefined aim was to investigate anaemia in general; respective inclusion criterion provided; no focus on IDA |
| Scott 2002  (16) | "To evaluate the efficacy of perioperative recombinant human erythropoietin (r-HuEPO, epoetin alfa) in stimulating haematopoiesis and reducing allogeneic blood transfusion requirements in major head and neck cancer surgery." | Pts. scheduled for major head and neck oncologic surgery the University of Iowa hospitals; above 18 years and Hb level between 10.0 g/dL and 13.5 g/dL, and have their surgical procedure scheduled at least 10 days from the date of potential study enrolment" | Head and neck surgery | Hb 10-13.5 mg/dl | NA | EPO + Iron oral | Iron oral | Change in Hb | Predefined aim was to investigate anaemia in general; respective inclusion criterion provided; no focus on IDA |

| *Additional file 2. continued* | | | | | | | | | |
| --- | --- | --- | --- | --- | --- | --- | --- | --- | --- |
| **Study ID** | **Rationale/aim/idea of the study, cited from study** | **Investigated population according to study** | **Type of surgery** | **Definition of anemia as inclusion criterion by study** | **Definition of iron deficiency as inclusion criterion by study** | **Inter-vention** | **Compa-rator** | **Primary outcome according to study** | **Assessment** |
| Tahir 2019  (17) | "To compare the efficacy and safety of subcutaneously administered recombinant human erythropoietin in combination with intravenous iron sucrose versus intravenous sucrose only for the management of iron deficiencyanaemia in gynaecological patients waiting for surgery" | Pts. with iron deficiency anaemia | Gynaecological surgery | mean haemoglobin level 7 g/dl | NA | EPO + Iron IV | Iron IV | Hb at day 14 after surgery | There is explicit claim that the study included iron-deficient anaemic. However, there is lack of detailed definition of inclusion criteria regarding anaemia or iron deficiency study |
| Urena 2017  (18) | “The aim of this study was to evaluate, in anaemic patients, the efficacy of EPO in reducing red cell transfusion rates post TAVI” | Pts. above 60 years and indication for TAVI with anaemia according to WHO 13.0 g/dl | Cardiac surgery | Hb <12 (f) or  13 (m) g/dl | NA | EPO + Iron oral | Placebo | Transfusion rate | Predefined aim was to investigate anaemia in general; respective inclusion criterion provided; no focus on IDA |

| *Additional file 2. continued* | | | | | | | | | |
| --- | --- | --- | --- | --- | --- | --- | --- | --- | --- |
| **Study ID** | **Rationale/aim/idea of the study, cited from study** | **Investigated population according to study** | **Type of surgery** | **Definition of anemia as inclusion criterion by study** | **Definition of iron deficiency as inclusion criterion by study** | **Inter-vention** | **Compa-rator** | **Primary outcome according to study** | **Assessment** |
| Weber 2005  (19) | “This study examined the impact of preoperative epoetin alfa administration on postoperative recovery and infection rate” | Pts. scheduled  for elective major orthopaedic surgery (hip, knee or  spine; primary or revision) and with a preoperative  Hb concentration between 10 and 13 g/dl | Orthopaedic surgery | Hb 10-13 mg/dl | NA | EPO + Iron oral | No treatment or iron IV/oral according to local standard of care | Change in Hb; transfusion rate | Predefined aim was to investigate anaemia in general; respective inclusion criterion provided; no focus on IDA |
| EPO: erythropoetin; f: female; Hb: hemoglobin; IDA: iron deficiency anemia; Iron IV: intravenous iron; m: male; NA: not applicable; Pts: patients; TAVI: transcatheter aortic-valve implantation; | | | | | | | | | |

**References**

1. Biboulet P, Bringuier S, Smilevitch P, Loupec T, Thuile C, Pencole M, et al. Preoperative Epoetin-α with Intravenous or Oral Iron for Major Orthopedic Surgery: A Randomized Controlled Trial. Anesthesiology. 2018;129(4):710-20.

2. Bielza R, Llorente J, Thuissard IJ, Andreu-Vázquez C, Blanco D, Sanjurjo J, et al. Effect of intravenous iron on functional outcomes in hip fracture: a randomised controlled trial. Age Ageing. 2021;50(1):127-34.

3. Buljan M, Nemet D, Golubic-Cepulic B, Bicanic G, Tripkovic B, Delimar D. Two different dosing regimens of human recombinant erythropoietin beta during preoperative autologous blood donation in patients having hip arthroplasty. International Orthopaedics. 2012;36(4):703-9.

4. Christodoulakis M, Tsiftsis DD. Preoperative Epoetin Alfa in Colorectal Surgery: A Randomized, Controlled Study. Annals of Surgical Oncology. 2005;12(9):718-25.

5. Dousias V, Paraskevaidis E, Dalkalitsis N, Tsanadis G, Navrozoglou I, Lolis D. Recombinant human erythropoietin in mildly anemic women before total hysterectomy. Clin Exp Obstet Gynecol. 2003;30(4):235-8.

6. Edwards TJ, Noble EJ, Durran A, Mellor N, Hosie KB. Randomized clinical trial of preoperative intravenous iron sucrose to reduce blood transfusion in anaemic patients after colorectal cancer surgery. Br J Surg. 2009;96(10):1122-8.

7. Kateros K, Sakellariou VI, Sofianos IP, Papagelopoulos PJ. Epoetin alfa reduces blood transfusion requirements in patients with intertrochanteric fracture. Journal of Critical Care. 2010;25(2):348-53.

8. Keeler BD, Simpson JA, Ng O, Padmanabhan H, Brookes MJ, Acheson AG. Randomized clinical trial of preoperative oral versus intravenous iron in anaemic patients with colorectal cancer. Br J Surg. 2017;104(3):214-21.

9. Kosmadakis N, Messaris E, Maris A, Katsaragakis S, Leandros E, Konstadoulakis MM, et al. Perioperative Erythropoietin Administration in Patients With Gastrointestinal Tract Cancer: Prospective Randomized Double-Blind Study. Annals of Surgery. 2003;237(3):417-21.

10. Larson B, Bremme K, Clyne N, Nordström L. Preoperative treatment of anemic women with epoetin beta. Acta Obstet Gynecol Scand. 2001;80(6):559-62.

11. Lidder PG, Sanders G, Whitehead E, Douie WJ, Mellor N, Lewis SJ, et al. Pre-operative oral iron supplementation reduces blood transfusion in colorectal surgery - a prospective, randomised, controlled trial. Ann R Coll Surg Engl. 2007;89(4):418-21.

12. M. MAFC, W. TBJ, T. KN, J. vOJ, D. VA, P. P. Pre-operative injections of epoetin-α versus post-operative retransfusion of autologous shed blood in total hip and knee replacement. The Journal of Bone and Joint Surgery British volume. 2008;90-B(8):1079-83.

13. Phipps O, Al-Hassi HO, Quraishi MN, Dickson EA, Segal J, Steed H, et al. Oral and Intravenous Iron Therapy Differentially Alter the On- and Off-Tumor Microbiota in Anemic Colorectal Cancer Patients. Cancers (Basel). 2021;13(6).

14. Richards T, Baikady RR, Clevenger B, Butcher A, Abeysiri S, Chau M, et al. Preoperative intravenous iron to treat anaemia before major abdominal surgery (PREVENTT): a randomised, double-blind, controlled trial. Lancet. 2020;396(10259):1353-61.

15. Rosencher N, Poisson D, Albi A, Aperce M, Barré J, Samama CM. Two injections of erythropoietin correct moderate anemia in most patients awaiting orthopedic surgery. Canadian Journal of Anesthesia. 2005;52(2):160-5.

16. Scott SN, Boeve TJ, McCulloch TM, Fitzpatrick KA, Karnell LH. The Effects of Epoetin Alfa on Transfusion Requirements in Head and Neck Cancer Patients: A Prospective, Randomized, Placebo-Controlled Study. The Laryngoscope. 2002;112(7):1221-9.

17. TAHIR S, SAEED K, NAZEER S. Comparison of Intravenous Iron Sucrose alone Versus Intravenous Iron Sucrose Along With Erythropoietin for Management of Anemia for Gynecological Patients Waiting for Surgery.

18. Marina U, Maria Del T, Omar Abdul-Jawad A, Francisco C-P, Ander R, Robert De L, et al. Combined erythropoietin and iron therapy for anaemic patients undergoing transcatheter aortic valve implantation: the EPICURE randomised clinical trial. EuroIntervention. 2017;13(1):44-52.

19. Weber EWG, Slappendel R, Hémon Y, Mähler S, Dalén T, Rouwet E, et al. Effects of epoetin alfa on blood transfusions and postoperative recovery in orthopaedic surgery: the European Epoetin Alfa Surgery Trial (EEST). European Journal of Anaesthesiology | EJA. 2005;22(4):249-57.

20. Abdullah HR, Thamnachit T, Hao Y, Lim WY, Teo LM, Sim YE. Real-world results of the implementation of preoperative anaemia clinic with intravenous iron therapy for treating iron-deficiency anaemia: a propensity-matched case-control study. Annals of Translational Medicine. 2020;9(1):6.

21. D'Amato T, Kon E, Martorelli F, Monteleone G, Simili V, Tasso F, et al. Effect of intravenous ferric carboxymaltose supplementation in non-anaemic iron deficient patients undergoing hip and knee arthroplasty. J Biol Regul Homeost Agents. 2020;34(4 Suppl. 3):69-77. Congress of the Italian Orthopaedic Research Society.

22. Froessler B, Palm P, Weber I, Hodyl NA, Singh R, Murphy EM. The Important Role for Intravenous Iron in Perioperative Patient Blood Management in Major Abdominal Surgery: A Randomized Controlled Trial. Annals of Surgery. 2016;264(1).

23. Ionescu A, Sharma A, Kundnani NR, Mihăilescu A, David VL, Bedreag O, et al. Intravenous iron infusion as an alternative to minimize blood transfusion in peri-operative patients. Scientific Reports. 2020;10(1):18403.

24. Kim YH, Chung HH, Kang SB, Kim SC, Kim YT. Safety and Usefulness of Intravenous Iron Sucrose in the Management of Preoperative Anemia in Patients with Menorrhagia: A Phase IV, Open-Label, Prospective, Randomized Study. Acta Haematologica. 2009;121(1):37-41.

25. Klein AA, Chau M, Yeates JA, Collier T, Evans C, Agarwal S, et al. Preoperative intravenous iron before cardiac surgery: a prospective multicentre feasibility study. British Journal of Anaesthesia. 2020;124(3):243-50.

26. Laso-Morales M, Jericó C, Gómez-Ramírez S, Castellví J, Viso L, Roig-Martínez I, et al. Preoperative management of colorectal cancer–induced iron deficiency anemia in clinical practice: data from a large observational cohort. Transfusion. 2017;57(12):3040-8.

27. Lee S, Ryu K-J, Lee ES, Lee KH, Lee JJ, Kim T. Comparative efficacy and safety of intravenous ferric carboxymaltose and iron sucrose for the treatment of preoperative anemia in patients with menorrhagia: An open-label, multicenter, randomized study. Journal of Obstetrics and Gynaecology Research. 2019;45(4):858-64.

28. Na H-S, Shin S-Y, Hwang J-Y, Jeon Y-T, Kim C-S, Do S-H. Effects of intravenous iron combined with low-dose recombinant human erythropoietin on transfusion requirements in iron-deficient patients undergoing bilateral total knee replacement arthroplasty (CME). Transfusion. 2011;51(1):118-24.

29. Nandhra S, Chau M, Klein AA, Yeates JA, Collier T, Evans C, et al. Preoperative anaemia management in patients undergoing vascular surgery. Br J Surg. 2020;107(12):1558-61.

30. Scardino M, Di Matteo B, Martorelli F, Tanzi D, Kon E, D’Amato T. Improved patient blood management and cost saving in hip replacement surgery through the implementation of pre-operative Sucrosomial® iron supplementation: a quality improvement assessment study. International Orthopaedics. 2019;43(1):39-46.

31. Shin K-H, Park J-H, Jang K-M, Hong S-H, Han S-B. Effects of intravenous iron monotherapy for patients with iron deficient anemia undergoing total knee arthroplasty. Arthroplasty. 2020;2(1):22.

32. Thin TN, Tan BPY, Sim EY, Shum KL, Chan HSP, Abdullah HR. Preoperative Single-Dose Intravenous Iron Formulation to Reduce Postsurgical Complications in Patients Undergoing Major Abdominal Surgery: A Randomized Control Trial Feasibility Study (PIRCAS Trial Pilot). Cureus. 2021;13(8):e17357.
