# Supplementary material for: Reported outcomes in patients with iron deficiency or iron deficiency anemia undergoing major surgery: a systematic review of outcomes
Source: Syst Rev. 2024 Jan 2;13:5. doi: 10.1186/s13643-023-02431-x (PMC10759584; doi:10.1186/s13643-023-02431-x)
Supplement: Supplementary file 4 — Additional file 4. Detailed study characteristics of eligible studies on iron deficiency, with or without anemia [file 13643_2023_2431_MOESM4_ESM.docx]

**Reported outcomes in patients with iron deficiency or iron deficiency anemia undergoing major surgery: a systematic review of outcomes**

**Additional file 4. Detailed study characteristics of eligible studies on iron deficiency, with or without anemia**

| **Study**  **[Registration number]** | **Country** | **Study type** | **Definition of anemia (Hb level)** | **Diagnostic criteria of iron deficiency** | **Intervention group (n)** | **Control group*** **(n)** | **Type of surgery** | **Recommendation on start of iron** | **Age, mean (**±**STD), yrs** |
| --- | --- | --- | --- | --- | --- | --- | --- | --- | --- |
| Abdullah-2021  (20) | Singapore | Retrospective cohort study | Hb <13 g/dl | Serum ferritin <100 mcg/L  and  transferrin saturation <20% | 89 iron deficiency anemia (IDA) patients receiving intravenous (**IV**) **iron**  Ferric carboxymaltose 15 mg/kg over 15 minutes (max 1000 mg) | 89 (1:1 matching) and 178 (1:2 matching), respectively, IDA patients receiving **active comparator**  Oral iron (not specified) | Elective surgeries requiring general anesthesia or regional anesthesia, except for transplant, burns and cardiac surgery | One month according to institutional protocol, in case postponing surgery is possible | Intervention group:  55.2 (±15.3),  Control group:  54.8 (±17.9) (1:1 matched) |
| D'Amato-2020  (21) | Italy | Retrospective cohort study | NA – only non-anemic ID patients included with  Hb ≥13 and ≤14 g/dl for men and ≥12 and ≤14 g/dl for women | Serum ferritin <100 μg/L and  normal CRP values | 83 iron deficiency (ID) patients receiving **IV iron**  Single infusion ferric carboxymaltose (1000mg over 15 minutes) | 62 ID patients receiving standard care (**SoC**)  No preoperative iron | Total hip arthroplasty or total knee arthroplasty | Four weeks prior to surgery | Intervention group:  67.6 (±9.5)  Control group:  66.0 (±10.9) |

| *Additional file 4. continued* | | | | | | | | | |
| --- | --- | --- | --- | --- | --- | --- | --- | --- | --- |
| **Study**  **[Registration number]** | **Country** | **Study type** | **Definition of anemia (Hb level)** | **Diagnostic criteria of iron deficiency** | **Intervention group (n)** | **Control group*** **(n)** | **Type of surgery** | **Recommendation on start of iron** | **Age, mean (**±**STD), yrs** |
| Froessler-2016  (22)  [ACTRN12611000387921] | Australia | RCT – Phase III | Hb <130 g/L in men and <120 g/L in women | Ferritin <300 mcg/L, transferrin saturation <25% | 40 IDA patients receiving **IV iron**  single dose ferric carboxymaltose  preoperative dose: 15 mg/kg or max. 1000 mg;  postoperative dose: 0.5 mg per 1 ml estimated blood loss (if blood loss was at least 100 mL) | 32 IDA patients receiving **SoC:**  perioperative care, including anemia management, provided by the primary care physician or surgical home team. Usual care provided included no treatment, continued observations, oral iron recommendations, and allogenic blood transfusion. At the time of initiation of the study, IV iron was not considered usual care; however, prescription and administration was not disallowed;  in SoC group n=3 received oral iron; n=5 received IV iron | Major abdominal surgery | 21 to 4 days before surgery (and within 2 days after surgery) | Intervention group:  64 (±15)  Control group:  68 (±15) |

| *Additional file 4. continued* | | | | | | | | | |
| --- | --- | --- | --- | --- | --- | --- | --- | --- | --- |
| **Study**  **[Registration number]** | **Country** | **Study type** | **Definition of anemia (Hb level)** | **Diagnostic criteria of iron deficiency** | **Intervention group (n)** | **Control group*** **(n)** | **Type of surgery** | **Recommendation on start of iron** | **Age, mean (**±**STD), yrs** |
| Ionescu-2020  (23) | Romania | Retrospective cohort study | Hb <130 g/L in men and <120 g/L in women | Ferritin < 300mcg/l, transferrin saturation <25% | 329 IDA patients receiving **IV iron:**  Ferric carboxymaltose  Dose: 10 ml/500 mg vials and a maximum dose of 20 ml/1000 mg was administered per week | 342 IDA patients receiving **active comparator**: allogenic blood transfusion;  2,282 IDA patients receiving **SoC:**  no iron supplementation, no allogenic blood transfusion | Surgery (not specified) | Not reported | NA |
| Kim-2009  (24) | South Korea | RCT –  Phase IV | Hb levels <9.0 g/dl (as inclusion criteria) | No criteria provided (*established IDA* as inclusion criteria) | 39 patients with established IDA receiving **IV iron**  Iron sucrose every other day, 3 times per week  dose calculated by formula: weight (kg) [target Hb (g/dl) – actual Hb (g/ dl)] 2.4 + 500 mg, and rounded up to the nearest multiple of 100 mg (max. total dose 200mg in 100ml NaCl) | 37 patients with established IDA receiving **active comparator**  Oral iron protein succinylate | Gynecological surgery (not specified) | Both treatments (IV and oral iron) starting 3 weeks before surgery | Intervention group:  42.0 (±7.4)  Control group:  42.3 (±8.0) |

| *Additional file 4. continued* | | | | | | | | | |
| --- | --- | --- | --- | --- | --- | --- | --- | --- | --- |
| **Study**  **[Registration number]** | **Country** | **Study type** | **Definition of anemia (Hb level)** | **Diagnostic criteria of iron deficiency** | **Intervention group (n)** | **Control group*** **(n)** | **Type of surgery** | **Recommendation on start of iron** | **Age, mean (**±**STD), yrs** |
| Klein-2020  (25)  [NCT02637102], [ISRCTN55032357] | UK | Prospective cohort study  (CAVIAR Study) | Hb <130 g/L in men and <120 g/L in women | Ferritin <100 *µg /L* and transferrin saturation <20% | 68 IDA patients receiving **IV iron**  Single dose iron isomaltoside 1000 at a total dose of calculated at 20 mg/kg or ferric carboxymaltose to a maximum of 1000 mg; both over at least 15–30 minutes | 75 IDA patients receiving **SoC**  No IV iron treatment | Cardiac surgery | At least 10 days before surgery | Intervention group:  70.2 (±10.9)  Control group:  69.3 (±11.8) |
| Laso- Morales-2017  (26) | Spain | Retrospective cohort study | Hb <13 g/dL; | Serum ferritin <30 ng/mL;  anemic patients with inflammation: CRP >5mg/L, transferrin saturation <20% with normal-to-elevated ferritin levels (30-500 ng/mL) | 232 IDA patients receiving **IV iron**  Iron sucrose: doses of 200 mg in 100 mL saline over 30 to 60 minutes;  or ferric carboxymaltose: 500 to 1000 mg in 200 mL saline over 15 to 30 minutes, once a week  preoperatively | 90 IDA patients receiving **SoC:**  No iron (n=67) treatment or oral iron (n=23) (100 mg elemental  iron) | Colorectal cancer resection | 6 to 4 weeks before surgery patients were referred to anemia clinic | Intervention group: 71 (±11)  Control group:  69 (±15) |

| *Additional file 4. continued* | | | | | | | | | |
| --- | --- | --- | --- | --- | --- | --- | --- | --- | --- |
| **Study**  **[Registration number]** | **Country** | **Study type** | **Definition of anemia (Hb level)** | **Diagnostic criteria of iron deficiency** | **Intervention group (n)** | **Control group*** **(n)** | **Type of surgery** | **Recommendation on start of iron** | **Age, mean (**±**STD), yrs** |
| Lee-2019  (27) | South Korea | RCT – Phase NA | Hb level <10 g/dL | Serum ferritin <30 ng/mL | 52 IDA patients receiving **IV iron**  Single dose ferric  carboxymaltose (<50 kg, 500 mg iron; ≥50 kg, 1000 mg iron) | 49 IDA patients receiving **active comparator**  IV iron  sucrose (dosage according to Ganzoni formula, max 600mg in 3 doses per week à 200mg) | Gynecological surgery for benign gynecological diseases causing menorrhagia | Not specified – probably 2 weeks before surgery | Intervention group:  44.0 (±5.7)  Control group:  43.4 (±5.0) |
| Na-2011  (28) | South Korea | RCT – Phase NA | NA –  only patients with Hb >100 g/L were included | Serum ferritin <100 ng/mL  or  ferritin 100 - 300 ng/mL with a transferrin saturation <20% | 56 IDA patients receiving **IV iron** with **EPO**  200 mg of iron sucrose iv + 3000 IU of rHuEPO-b sc (once during surgery and administered repeatedly when Hb on postop days 1, 2, 3, and 5 was 70 to 80 g/L) | 57 IDA patients receiving **SoC**  No treatment (not specified) | Bilateral total knee replacement | NA –  first does perioperative | Intervention group:  69.4 (±4.1)  Control group:  67.9 (±5.2) |
| Nandhra-2020  (29)  [NCT02637102], [ISRCTN55032357] | UK | Prospective cohort study  (CAVIAR study) | Hb <130 g/L in men and <120 g/L in women | Ferritin level <100 μg/l and/or transferrin saturation <20% | 15 IDA patients receiving **IV iron**  (not specified in publication) | 57 IDA patients receiving **SoC**  No treatment | Vascular surgery | At least 10 days before surgery | Intervention group:  71.8 (±7.1)  Control group:  74.4 (±9.0) |

| *Additional file 4. continued* | | | | | | | | | |
| --- | --- | --- | --- | --- | --- | --- | --- | --- | --- |
| **Study**  **[Registration number]** | **Country** | **Study type** | **Definition of anemia (Hb level)** | **Diagnostic criteria of iron deficiency** | **Intervention group (n)** | **Control group*** **(n)** | **Type of surgery** | **Recommendation on start of iron** | **Age, mean (**±**STD), yrs** |
| Scardino-2019  (30) | Italy | Retrospective cohort study | NA – anemia was exclusion criteria | Ferritin <100 mcg/l or ferritin >100 mcg/l but CRP >3 mg/l and transferrin saturation <20% | 100 ID patients receiving **oral iron**  Sucrosomial® iron, 30 mg (capsule) daily | 100 ID patients receiving **SoC**  No iron treatment | Hip surgery | Three to four weeks prior to surgery | Intervention group:  68.8 (±9.4)  Control group:  68.4 (±9.5) |
| Shin-2020  (31) | South Korea | Prospective cohort study with historic control | Hb levels <12.0 g/dL for women and <13.0 g/dL for men | Intervention group: ferritin <30 μg/L or 30–100 μg/L and transferrin saturation <20%;  Control group: not tested | 46 IDA patients receiving **IV iron**  Single dose ferric carboxymaltose (1000mg) | 46 (propensitiy score matched) non-ID-tested, anemic patients receiving **SoC**  No iron treatment | Total knee arthroplasty | Four weeks prior to surgery | Intervention group:  67.71 (±7.94)  Control group:  69.82 (±5.38) (propensity score matched) |
| Thin-2021  (32)  [NCT03295851] | Singapore | RCT – Phase IV (pilot study)  (PIRCAS trial) | Hb <13.0 g/dL in men and <12.0 g/dL in women | Ferritin <100 µg/L or ferritin 100 µg/L - 300 µg/L, transferrin saturation <20% | 15 IDA patients receiving IV iron  Single dose ferric carboxymaltose 15 mg/kg or up to 1000 mg | 15 IDA patients receiving **active comparator**  Oral ferrous fumarate, 200 mg twice daily | Major abdominal surgery | IV iron: 4 weeks prior to surgery  Oral iron: 1-4 weeks before surgery | Intervention group:  59.2 (±12.4),  Control group:  55.2 (±23.3) |
| **Abbreviations:** CRP: C-reactive protein; EPO: erythropoetin-b; Hb: hemoglobin; IV iron: intravenous iron; n: number of participants; NA: not applicable; RCT: randomized controlled trial; SoC: standard of care; TSAT: transferrin serum iron saturation;  *Extracted information on control group restricted to patients with ID or IDA (except for Shin et al.) | | | | | | | | | |

**References**

1. Biboulet P, Bringuier S, Smilevitch P, Loupec T, Thuile C, Pencole M, et al. Preoperative Epoetin-α with Intravenous or Oral Iron for Major Orthopedic Surgery: A Randomized Controlled Trial. Anesthesiology. 2018;129(4):710-20.

2. Bielza R, Llorente J, Thuissard IJ, Andreu-Vázquez C, Blanco D, Sanjurjo J, et al. Effect of intravenous iron on functional outcomes in hip fracture: a randomised controlled trial. Age Ageing. 2021;50(1):127-34.

3. Buljan M, Nemet D, Golubic-Cepulic B, Bicanic G, Tripkovic B, Delimar D. Two different dosing regimens of human recombinant erythropoietin beta during preoperative autologous blood donation in patients having hip arthroplasty. International Orthopaedics. 2012;36(4):703-9.

4. Christodoulakis M, Tsiftsis DD. Preoperative Epoetin Alfa in Colorectal Surgery: A Randomized, Controlled Study. Annals of Surgical Oncology. 2005;12(9):718-25.

5. Dousias V, Paraskevaidis E, Dalkalitsis N, Tsanadis G, Navrozoglou I, Lolis D. Recombinant human erythropoietin in mildly anemic women before total hysterectomy. Clin Exp Obstet Gynecol. 2003;30(4):235-8.

6. Edwards TJ, Noble EJ, Durran A, Mellor N, Hosie KB. Randomized clinical trial of preoperative intravenous iron sucrose to reduce blood transfusion in anaemic patients after colorectal cancer surgery. Br J Surg. 2009;96(10):1122-8.

7. Kateros K, Sakellariou VI, Sofianos IP, Papagelopoulos PJ. Epoetin alfa reduces blood transfusion requirements in patients with intertrochanteric fracture. Journal of Critical Care. 2010;25(2):348-53.

8. Keeler BD, Simpson JA, Ng O, Padmanabhan H, Brookes MJ, Acheson AG. Randomized clinical trial of preoperative oral versus intravenous iron in anaemic patients with colorectal cancer. Br J Surg. 2017;104(3):214-21.

9. Kosmadakis N, Messaris E, Maris A, Katsaragakis S, Leandros E, Konstadoulakis MM, et al. Perioperative Erythropoietin Administration in Patients With Gastrointestinal Tract Cancer: Prospective Randomized Double-Blind Study. Annals of Surgery. 2003;237(3):417-21.

10. Larson B, Bremme K, Clyne N, Nordström L. Preoperative treatment of anemic women with epoetin beta. Acta Obstet Gynecol Scand. 2001;80(6):559-62.

11. Lidder PG, Sanders G, Whitehead E, Douie WJ, Mellor N, Lewis SJ, et al. Pre-operative oral iron supplementation reduces blood transfusion in colorectal surgery - a prospective, randomised, controlled trial. Ann R Coll Surg Engl. 2007;89(4):418-21.

12. M. MAFC, W. TBJ, T. KN, J. vOJ, D. VA, P. P. Pre-operative injections of epoetin-α versus post-operative retransfusion of autologous shed blood in total hip and knee replacement. The Journal of Bone and Joint Surgery British volume. 2008;90-B(8):1079-83.

13. Phipps O, Al-Hassi HO, Quraishi MN, Dickson EA, Segal J, Steed H, et al. Oral and Intravenous Iron Therapy Differentially Alter the On- and Off-Tumor Microbiota in Anemic Colorectal Cancer Patients. Cancers (Basel). 2021;13(6).

14. Richards T, Baikady RR, Clevenger B, Butcher A, Abeysiri S, Chau M, et al. Preoperative intravenous iron to treat anaemia before major abdominal surgery (PREVENTT): a randomised, double-blind, controlled trial. Lancet. 2020;396(10259):1353-61.

15. Rosencher N, Poisson D, Albi A, Aperce M, Barré J, Samama CM. Two injections of erythropoietin correct moderate anemia in most patients awaiting orthopedic surgery. Canadian Journal of Anesthesia. 2005;52(2):160-5.

16. Scott SN, Boeve TJ, McCulloch TM, Fitzpatrick KA, Karnell LH. The Effects of Epoetin Alfa on Transfusion Requirements in Head and Neck Cancer Patients: A Prospective, Randomized, Placebo-Controlled Study. The Laryngoscope. 2002;112(7):1221-9.

17. TAHIR S, SAEED K, NAZEER S. Comparison of Intravenous Iron Sucrose alone Versus Intravenous Iron Sucrose Along With Erythropoietin for Management of Anemia for Gynecological Patients Waiting for Surgery.

18. Marina U, Maria Del T, Omar Abdul-Jawad A, Francisco C-P, Ander R, Robert De L, et al. Combined erythropoietin and iron therapy for anaemic patients undergoing transcatheter aortic valve implantation: the EPICURE randomised clinical trial. EuroIntervention. 2017;13(1):44-52.

19. Weber EWG, Slappendel R, Hémon Y, Mähler S, Dalén T, Rouwet E, et al. Effects of epoetin alfa on blood transfusions and postoperative recovery in orthopaedic surgery: the European Epoetin Alfa Surgery Trial (EEST). European Journal of Anaesthesiology | EJA. 2005;22(4):249-57.

20. Abdullah HR, Thamnachit T, Hao Y, Lim WY, Teo LM, Sim YE. Real-world results of the implementation of preoperative anaemia clinic with intravenous iron therapy for treating iron-deficiency anaemia: a propensity-matched case-control study. Annals of Translational Medicine. 2020;9(1):6.

21. D'Amato T, Kon E, Martorelli F, Monteleone G, Simili V, Tasso F, et al. Effect of intravenous ferric carboxymaltose supplementation in non-anaemic iron deficient patients undergoing hip and knee arthroplasty. J Biol Regul Homeost Agents. 2020;34(4 Suppl. 3):69-77. Congress of the Italian Orthopaedic Research Society.

22. Froessler B, Palm P, Weber I, Hodyl NA, Singh R, Murphy EM. The Important Role for Intravenous Iron in Perioperative Patient Blood Management in Major Abdominal Surgery: A Randomized Controlled Trial. Annals of Surgery. 2016;264(1).

23. Ionescu A, Sharma A, Kundnani NR, Mihăilescu A, David VL, Bedreag O, et al. Intravenous iron infusion as an alternative to minimize blood transfusion in peri-operative patients. Scientific Reports. 2020;10(1):18403.

24. Kim YH, Chung HH, Kang SB, Kim SC, Kim YT. Safety and Usefulness of Intravenous Iron Sucrose in the Management of Preoperative Anemia in Patients with Menorrhagia: A Phase IV, Open-Label, Prospective, Randomized Study. Acta Haematologica. 2009;121(1):37-41.

25. Klein AA, Chau M, Yeates JA, Collier T, Evans C, Agarwal S, et al. Preoperative intravenous iron before cardiac surgery: a prospective multicentre feasibility study. British Journal of Anaesthesia. 2020;124(3):243-50.

26. Laso-Morales M, Jericó C, Gómez-Ramírez S, Castellví J, Viso L, Roig-Martínez I, et al. Preoperative management of colorectal cancer–induced iron deficiency anemia in clinical practice: data from a large observational cohort. Transfusion. 2017;57(12):3040-8.

27. Lee S, Ryu K-J, Lee ES, Lee KH, Lee JJ, Kim T. Comparative efficacy and safety of intravenous ferric carboxymaltose and iron sucrose for the treatment of preoperative anemia in patients with menorrhagia: An open-label, multicenter, randomized study. Journal of Obstetrics and Gynaecology Research. 2019;45(4):858-64.

28. Na H-S, Shin S-Y, Hwang J-Y, Jeon Y-T, Kim C-S, Do S-H. Effects of intravenous iron combined with low-dose recombinant human erythropoietin on transfusion requirements in iron-deficient patients undergoing bilateral total knee replacement arthroplasty (CME). Transfusion. 2011;51(1):118-24.

29. Nandhra S, Chau M, Klein AA, Yeates JA, Collier T, Evans C, et al. Preoperative anaemia management in patients undergoing vascular surgery. Br J Surg. 2020;107(12):1558-61.

30. Scardino M, Di Matteo B, Martorelli F, Tanzi D, Kon E, D’Amato T. Improved patient blood management and cost saving in hip replacement surgery through the implementation of pre-operative Sucrosomial® iron supplementation: a quality improvement assessment study. International Orthopaedics. 2019;43(1):39-46.

31. Shin K-H, Park J-H, Jang K-M, Hong S-H, Han S-B. Effects of intravenous iron monotherapy for patients with iron deficient anemia undergoing total knee arthroplasty. Arthroplasty. 2020;2(1):22.

32. Thin TN, Tan BPY, Sim EY, Shum KL, Chan HSP, Abdullah HR. Preoperative Single-Dose Intravenous Iron Formulation to Reduce Postsurgical Complications in Patients Undergoing Major Abdominal Surgery: A Randomized Control Trial Feasibility Study (PIRCAS Trial Pilot). Cureus. 2021;13(8):e17357.
